# Therapeutic strategy for exosome-based bone regeneration to osteoporosis: Challenges and potential solutions

**DOI:** 10.1016/j.jare.2025.09.042

**Published:** 2025-09-25

**Authors:** Anoop Puthiyoth Dayanandan, Alvin Bacero Bello, Yoshie Arai, Sang Jin Lee, Soo-Hong Lee

**Affiliations:** a Department of Biomedical Engineering, Dongguk University, Seoul 04620, Republic of Korea; b Biofunctional Materials, Division of Applied Oral Sciences and Community Dental Care, Faculty of Dentistry, The University of Hong Kong, 34 Hospital Road, Sai Ying Pun, PR China

**Keywords:** Osteoporosis, Exosomes, Bone regeneration, Mesenchymal stem cells, Exosome engineering, Bone-targeted delivery

## Abstract

**Background:**

Osteoporosis (OP) is a progressive bone disease marked by reduced bone mass and microarchitectural deterioration, thereby increasing fracture risk. Current therapies only partly restore bone quality and often cause side effects with long-term use. Recent studies highlight exosomes, a subtype of extracellular vesicles, as key regulators of bone remodeling with promising regenerative potential. However, their role in osteoporosis remains underexplored.

**Aim of review:**

This review aims to investigate how the established role of exosomes in bone regeneration may inform novel therapeutic strategies for osteoporosis. By linking general bone repair mechanisms with osteoporotic pathology, it evaluates the translational potential and limitations of exosome-based interventions.

**Key scientific concepts of review:**

Exosomes derived from mesenchymal stem cells, osteoblasts, osteoclasts, and macrophages regulate bone homeostasis by modulating osteogenesis, osteoclastogenesis, and immune responses. Their regenerative effects in non-osteoporotic settings are well documented. However, challenges such as exosome heterogeneity, rapid clearance, and poor targeting efficiency impede their direct application in osteoporosis. Recent bioengineering approaches, including osteogenic RNA or protein loading and surface modifications for bone targeting, show promise. Moreover, combining exosomes with immunomodulatory or synergistic therapies may further enhance their efficacy. Despite these advances, gaps persist in standardizing isolation techniques, ensuring batch consistency, and scaling up production for clinical use. This review consolidates current knowledge and outlines directions for adapting exosome-based bone regenerative strategies to osteoporosis treatment.

## Highlights

• Osteoporosis causes weak bones and high fracture risk.
• Exosomes have strong potential for bone regeneration.
• Translating exosomes to therapy faces biological challenges.
• Engineering exosomes can improve stability and targeting.
• Combining exosomes with drugs or immune therapies boosts efficacy.

## Graphical abstract

Schematic representation of exosome-based bioengineering strategies for OP therapy. The central portion depicts exosomes derived from various cell sources, including OB, OC, osteocytes, macrophages, and iPSCs. Three major engineering strategies are illustrated: (1) Surface modifications (top-left) enhance targeting and uptake of exosomes via the functionalization of receptors (e.g., RANK, CD44), peptides (DSS6, RGD), and ligands (RANKL, ALD); (2) Cell preconditioning (top-right) under hypoxic or inflammatory conditions promotes osteogenesis and inhibits osteoclastogenesis; and (3) Cargo loading (bottom-left) involves the incorporation of functional RNA species such as siRNAs (e.g., targeting RANK, MMP9), mRNAs (e.g., RUNX2, BMP2), and miRNAs (e.g., miR-21, miR-26a) to stimulate bone formation and reduce resorption. Additionally, immune modification strategies (bottom-right) involving regulatory T cell (Treg) and macrophage-derived exosomes modulate inflammation and suppress osteoclast activity. Together, these approaches offer a versatile and integrated approach to improving the therapeutic efficacy of exosomes in treating bone-related disorders. OP, Osteoporosis; OC, osteoclasts; OB, osteoblasts; iPSCs, induced pluripotent stem cells.

**Figure:**
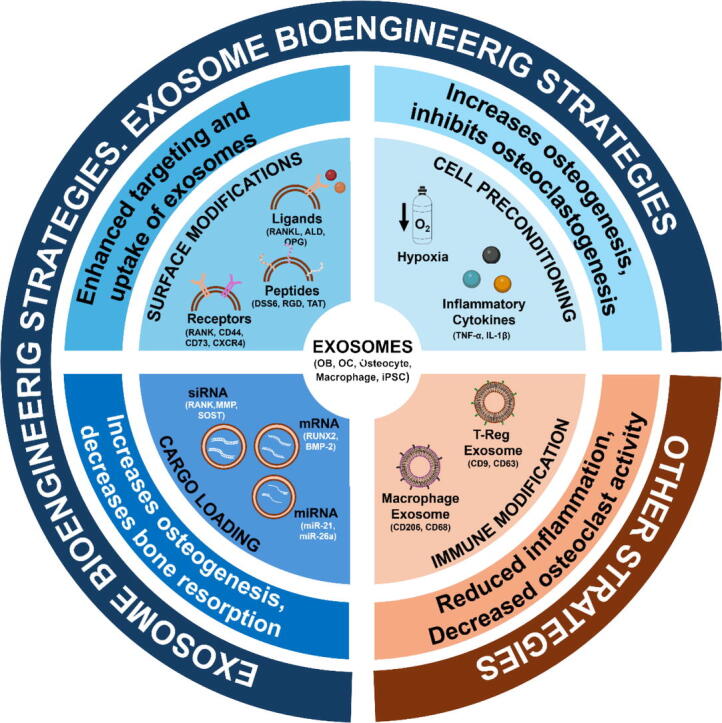

## Introduction

### Overview of osteoporosis

Osteoporosis (OP) is a chronic metabolic bone disease characterized by reduced bone strength and increased fracture risk [1,2]. Unlike other skeletal disorders, OP progresses silently and often remain undiagnosed until a fracture occurs, leading to significant morbidity and mortality [3,4]. It predominantly affects the elderly and postmenopausal women, driven by hormonal changes and age-related decline in bone regeneration [5,6]. With rising global life expectancy, OP has become a major public health challenge, recognized by the World Health Organization (WHO) as a leading cause of disability. Hip and vertebral fractures cause substantial healthcare costs, long-term disability, and increased mortality rates [7,8].

OP arises from disrupted bone remodeling, wherein osteoclast (OC)-driven bone resorption surpasses osteoblast (OB)-driven bone formation. This imbalance results from various factors, including hormonal fluctuations, oxidative stress, and inflammatory cytokines, that disrupt the tightly regulated bone microenvironment [[9], [10], [11]]. Additionally, age-related changes in the bone marrow niche, including impaired mesenchymal stem cell (MSC) differentiation and altered osteocyte signaling, further exacerbate bone loss [12,13]. These alterations reduce bone density and compromise bone quality, making osteoporotic bones prone to fractures even under minimal trauma.

One of the main factors for the deterioration of OP is the gut-bone axis where gut microbiota and their metabolites reshape bone remodeling through immune, endocrine, metabolic, and neurogenic routes. Microbial products such as short-chain fatty acids, bile-acid derivatives, and tryptophan metabolites can enhance osteoblastogenesis, restrain osteoclastogenesis, tighten gut barrier integrity, and rebalance Th17/Treg-skewed inflammation that fuels bone loss; conversely, dysbiosis promotes endotoxemia and cytokine signaling that tip remodeling toward resorption. Diet is a primary lever: fiber-, polyphenol-, and calcium/vitamin D supportive patterns remodel the microbiome toward metabolite profiles that favor bone accrual, while Westernized patterns have the opposite effect. Causality is strengthened by fecal microbiota transplantation studies in ovariectomized mice, where healthy-donor communities restore microbial function and prevent or reverse bone loss by normalizing metabolite pathways and inflammatory tone. Clinically tractable microbiome tools probiotics and prebiotics show promise as low-risk adjuncts, acting via the brain–gut–bone circuitry to reduce inflammatory signaling and improve bone indices in preclinical models (and emerging early human data). Together, these findings position microbiota-targeted strategies (metabolite-centric, dietary, microbial, or FMT-based) as complementary to bone-directed therapies, offering multi-node control over osteo-immunity and systemic factors that conventional drugs and local regenerative approaches may not fully address [[14], [15], [16], [17]].

Current OP treatments primarily rely on pharmacological agents that inhibit bone resorption or stimulate bone formation. These include antiresorptive agents such as bisphosphonates (BPs) and denosumab, which suppress OC activity to slow bone loss whereas anabolic therapies, including teriparatide and ramucirumab, enhance OB function to promote bone formation [18,19]. Although these treatments reduce fracture risk effectively, they present some limitations. Prolonged use of antiresorptive agents is associated with adverse effects, such as osteonecrosis of the jaw and atypical femoral fractures, while anabolic agents are often limited by high costs and restricted treatment duration. Although these agents effectively stimulate bone formation, they do not fully restore the bone microarchitecture and microenvironment, leading to incomplete and short-term therapeutic benefits [20,21].

### Need for new therapeutic strategies

The limitations of current OP therapies highlight the need for novel strategies to restore bone homeostasis and promote comprehensive bone regeneration. An ideal therapy would inhibit excessive bone resorption, stimulates new bone formation, and thereby restore the bone microenvironment while enhancing mechanical strength. The growing demand for safer and more effective long-term treatments drives the need for innovation that minimize adverse effects and improve patient adherence and outcomes.

Regenerative medicine has recently gained attention as a transformative approach for OP treatment aiming to prevent further bone loss while restoring bone structure and function. Central to this strategy, cell-based therapies use MSCs and other progenitors to promote bone regeneration through differentiation and paracrine signaling. Despite encouraging preclinical and early clinical results, these therapies face challenges, including poor engraftment, immune rejection, tumorigenicity risk, and complex regulatory barriers [[22], [23], [24]]. To address these limitations, research increasingly explores acellular strategies that harness the therapeutic potential of cell-secreted products. Among these, exosomes have emerged as promising candidates due to their pivotal role in intercellular communication and tissue repair. Exosomes, a sub-set of extracellular vesicle, are nanosized, membrane-bound vesicles actively secreted by various cell types and enriched with bioactive molecules [[25], [26], [27], [28]]. By delivering functional cargo to recipient cells, exosomes modulate key biological processes, such as osteogenesis, angiogenesis, and immunomodulation, positioning them as potent tools for next-generation regenerative therapies in OP.

Unlike conventional drugs, exosomes offer high biocompatibility, low immunogenicity, and intrinsic targeting, making them ideal candidates for bone regeneration [[29], [30], [31]]. Furthermore, exosomes derived from stem cells, OBs, and other bone-related cells enable the development of targeted therapies that modulate the bone microenvironment. Recent studies show that exosomes promote osteogenic differentiation, enhance bone matrix mineralization, and inhibit OC-mediated bone resorption, highlighting their dual role in bone formation and resorption [[32], [33], [34]]. For instance, MSC-derived exosomes have been shown to enhance osteogenic differentiation and mineralization, thereby supporting bone formation. For instance, exosomes from young MSCs have demonstrated the ability to promote bone regeneration in aged rats by enhancing the proliferation and osteogenic capacity of BMSCs [28]. Conversely, exosomes from aged MSCs, enriched with specific microRNAs, can suppress osteogenesis, highlighting the nuanced role of exosomes in bone metabolism. Beyond their osteogenic potential, exosomes also play a critical role in modulating OC activity. Studies have indicated that exosomes can influence osteoclastogenesis, thereby affecting bone resorption processes. This dual regulatory role underscores the complexity of exosome-mediated signaling in bone remodeling [27].

Beyond their inherent therapeutic properties, exosomes can be bioengineered to enhance stability, targeting, and therapeutic payload, advancing precision medicine. For example, surface modification of exosomes with bone-targeting ligands or incorporation of osteoinductive molecules enhance bone regeneration in preclinical OP models [[35], [36], [37]]. These advances suggest that exosome-based therapies could overcome the limitations of traditional treatments, offering a more comprehensive approach to OP management.

As evidence of their role in regenerative potential grows, exosomes are emerging as a promising frontier in OP therapy and regenerative medicine. However, despite progress in understanding exosome-mediated bone repair, their specific role in osteoporotic bone remains unclear. Therefore, this review aims to bridge this gap by exploring the therapeutic potential of exosomes in OP based on their established roles in bone regeneration. It examines how exosomes regulate bone homeostasis, their contributions from diverse cell sources-including OBs, OCs, MSCs, osteocytes, and macrophages and how these insights may translate into effective OP treatments. Additionally, the review addresses challenges in exosome-based therapies, such as biological variability, delivery strategies, and technical limitations, while exploring innovation such as engineered exosomes, RNA-loaded exosomes, and combinatorial therapies integrating immunomodulation or biomaterials. By synthesizing current knowledge and emerging strategies, this review seeks to provide a comprehensive framework for exosome-based therapeutics in OP, guiding future research and clinical translation.

## Rationale for using exosomes in bone regeneration

### Exosomes as natural delivery systems

Exosomes are small extracellular vesicles, typically 30–150  nm in diameter, of endosomal origin, released when multivesicular bodies fuse with the plasma membrane [38,39,[80], [81], [82], [83]]. Secreted by nearly all cell types, they contain a rich cargo of bioactive molecules, such as proteins, lipids, RNA, and DNA, and act as intercellular messengers that regulate various biological processes [[40], [41], [42]]. Their ability to target specific tissues, including bone, highlights their potential as natural delivery systems. Exosome composition depends on the cell of origin and the physiological conditions at release. For instance, MSC-derived exosomes are enriched with growth factors, cytokines, and osteogenic miRNAs that directly enhance bone formation and osteogenic differentiation [43,44].

The lipid bilayer membrane of the exosome protects its cargo from enzymatic degradation, ensuring stable delivery of bioactive molecules to target cells even in harsh environments such as acidic conditions at bone resorption sites [45].

### Advantages of exosome therapies

Exosome-based therapies offer key benefits over conventional bone regeneration treatments:

• **Superior efficacy**: Unlike systemically administered drugs with poor bone targeting, exosomes, particularly those from MSCs, possess inherent homing abilities. This enables precise delivery of therapeutic molecules to bone remodeling sites, enhancing efficacy while reducing dosage requirements [46,47].
• **Reduced side effects**: Conventional therapies, including antiresorptive (e.g., BPs and denosumab) and anabolic (e.g., teriparatide, abaloparatide, and romosozumab) agents, frequently cause off-target effects and systemic toxicity. For instance, postmenopausal selective estrogen receptor modulators (SERMs), such as raloxifene, reduce radiographically confirmed vertebral fractures by approximately 30–50 % over three years (e.g., data from the MORE trial summarized in benefit–risk analyses), but they have minimal or no effect on hip and other non-vertebral fractures. The primary safety concern is a roughly twofold increased risk of venous thromboembolism (VTE), with most events occurring during the first year of therapy (*meta*-analysis pooled OR ≈ 1.6–2.0) [[50], [51], [52]].

Calcitonin demonstrates modest efficacy. The five-year PROOF randomized controlled trial (n = 1,255) showed that 200 IU of intranasal salmon calcitonin daily reduced new vertebral fractures by 33 % versus placebo, while higher doses (400 IU) did not provide additional benefits. These studies also reported analgesic effects, with reduced back pain in patients with osteoporotic vertebral fractures. No significant reduction in non-vertebral fracture risk was observed [[53], [54], [55], [56], [57]]. Hormone replacement therapy (HRT) remains highly effective for vasomotor symptom management. Systemic estrogen therapy typically decreases hot-flash frequency and severity by 65–90 % according to randomized trials and guideline syntheses. However, combined estrogen-progestogen therapy carries an elevated breast cancer risk (relative risk ≈ 1.6 after 1–4 years of use, increasing with duration), whereas estrogen-only regimens generally show smaller or neutral effects. Transdermal administration is associated with a lower VTE risk compared with oral formulations [[58], [59], [60], [61], [62], [63]].

Among anabolic and antiresorptive biologics, teriparatide increases lumbar-spine bone mineral density (BMD) by approximately 9–13 % over 18 months and reduces new vertebral fractures by around 65 %, as shown in landmark RCTs. Denosumab, based on real-world Korean post-marketing surveillance data, significantly increased spine and hip BMD over 12 months without new safety concerns, consistent with phase 3 trial findings; a *meta*-analysis of roughly 22,000 patients indicated a slightly higher risk of serious infections versus placebo, although not exceeding that of active comparators. Romosozumab administered for 12 months increased lumbar-spine BMD by 11–13 % and total-hip BMD by 4–7 %, reducing new vertebral fractures by 73 % versus placebo (FRAME trial) and by 48 % versus alendronate over 24 months when followed by alendronate (ARCH trial). Cardiovascular safety signals were neutral in FRAME but slightly elevated in ARCH, warranting caution in labeling [52,[64], [65], [66], [67], [68], [69], [70], [71], [72]] (Table 1). Overall, traditional therapies such as SERMs, calcitonin, and HRT provide modest or site-specific benefits but are limited by safety concerns including VTE and breast cancer risk. In contrast, newer biologics like teriparatide, denosumab, and romosozumab offer substantial gains in bone density and vertebral fracture reduction, though long-term monitoring for infection and cardiovascular risks remains essential. Exosomes, due to their biocompatibility and low immunogenicity, mitigate these risks and are suitable for long-term use [73].

• **Potential for personalization:** Exosomes can be tailored to individual treatment needs, enabling personalized therapy [74]. They can be engineered to deliver specific osteogenic molecules or miRNAs tailored to’the unique bone regeneration requirements of each patient and modified to target precise cell types, enhancing therapeutic precision and effectiveness [75,76]. This versatility underscores their promise for precision medicine in OP treatment.

**Table 1:** **Summary of current pharmacological therapies for osteoporosis: mechanisms, benefits, limitations, and clinical considerations**. This table provides an overview of major therapeutic classes used for OP management, including representative agents, primary mechanisms of action, key advantages, limitations, common side effects, and typical patient populations. Reference numbers correspond to relevant supporting literature.

**Therapy Type**	**Examples**	**Mechanism of Action**	**Advantages**	**Limitations**	**Common Side Effects**	**Patient Population**	**Refs.**
Bisphosphonates	Alendronate, Risedronate, Ibandronate	Inhibit OC-mediated bone resorption, leading to increased bone mass.	Effective in reducing fracture risk, long history of use.	Risk of atypical femoral fractures and osteonecrosis of the jaw.	GI disturbances, flu-like symptoms, hypocalcemia.	Postmenopausal women, older adults.	[48,49]
Selective Estrogen Receptor Modulators (SERMs)	Raloxifene	Mimic estrogen effects on the bone to reduce resorption and increase bone density.	Reduced breast cancer risk, protective against vertebral fractures.	Limited effect on non-vertebral fractures.	Hot flashes, leg cramps, increased risk of deep vein thrombosis (DVT).	Postmenopausal women with osteoporosis.	[50,51]
Calcitonin	Calcitonin salmon	Inhibits OC activity and promotes bone formation.	Nasal spray option available, analgesic effects on fractures.	Limited effectiveness in preventing fractures.	Nasal irritation, allergic reactions.	Postmenopausal women; those intolerant to bisphosphonates.	[53,54]
Hormone Replacement Therapy (HRT)	Estrogen, Combined HRT	Restores estrogen levels, which help maintain bone density.	Effective in reducing fracture risk and improving menopause symptoms.	Increased risk of cardiovascular events and breast cancer.	Nausea, headaches, mood changes.	Postmenopausal women.	[[58], [59], [60]]
Parathyroid Hormone (PTH) Analogues	Teriparatide, Abaloparatide	Stimulates OB activity, increasing bone formation.	Significant increase in bone density and reduced fracture risk.	Limited duration of use (max 2 years).	Nausea, dizziness, leg cramps.	Severe OP, high fracture risk.	[64,65]
Denosumab	Denosumab (Prolia)	Monoclonal antibody that inhibits RANKL, reducing OC formation.	Effective in reducing fracture risk in various populations.	Risk of hypocalcemia and possible osteonecrosis of the jaw.	Back pain, musculoskeletal pain, infections.	Postmenopausal women, men with prostate cancer.	[52,68]
Romosozumab	Romosozumab	Sclerostin inhibitor that promotes bone formation while reducing resorption.	Significant increase in bone mass and strength.	Increased risk of cardiovascular events.	Injection site reactions, headache.	Postmenopausal women with high fracture risk.	[68,69]

## Exosomes in bone regeneration: mechanisms and roles

### Exosomes derived from different cell types

Msc-derived exosomes promote OB differentiation and enhance the bone-forming capacity of surrounding cells. OBs contribute to bone matrix formation by secreting exosomes that regulate OC activity and promote osteogenesis while OCs secrete exosomes that regulate bone resorption and maintain homeostasis. Osteocytes as mechanosensitive bone cells secrete exosomes that influence bone remodeling in response to mechanical stress [[77], [78], [79], [80]]. Macrophages essential regulators of the immune response secrete exosomes that modulate inflammation and influence osteoclastogenesis and osteogenesis. Furthermore induced pluripotent stem cells (iPSCs) are a novel source of exosomes with potential for regenerative therapies by promoting osteogenesis and tissue repair [81,82]. Collectively these exosomes forms intricate signaling networks that regulate bone formation and homeostasis, highlighting their therapeutic potential in bone diseases such as OP.

### Mesenchymal stem cell-derived exosomes

MSCs drive bone regeneration by differentiating into OBs and secreting bioactive factors that support bone repair and remodeling. However, limited cell survival and immune rejection have shifted attention to MSC-derived exosomes, including exosomes and microvesicles. They promote osteogenesis by delivering osteoinductive factors to target cells. Compared to MSCs, exosomes are more stable, less immunogenic, and lack uncontrolled proliferation, making them a promising alternative for bone regeneration therapies [[83], [84], [85], [86], [87], [88], [89], [90]].

Studies show that MSC-derived exosomes regulate osteogenic gene expression and enhance differentiation. For instance, bone marrow stromal cell (BMSC)-derived exosomes deliver osteogenic miRNAs, such as miR-196a, to OB, promoting *in vivo* bone formation [91]. Similarly, dental pulp stem cell (DPSC)-derived exosomes combined with scaffolds significantly enhance bone regeneration in rat calvarial defects [92]. Exosomes from osteo-induced MSCs exhibit enhanced osteogenic potential due to their enrichment in bone-related proteins sucha as WNT5A, NOTCH2, BMP2, SPP1, BSP, and BGLAP which promoted OB differentiation and calcium deposition [93]. These findings suggest that MSC-derived exosomes act as potent paracrine regulators that transfer osteo-inductive miRNAs and bone-related proteins to OBs, thereby reprogramming their gene expression and activating key signaling pathways to accelerate differentiation and bone regeneration.

MSC derived exosomes are critical mediators of angiogenesis an essential process for effective bone regeneration. For example exosomes from gingival MSCs enriched with osteogenesis-related genes and proteins such as ALP, OCN and RUNX2 enhanced osteogenesis in bone marrow MSCs and angiogenesis in endothelial cells thereby promoting bone formation and vascularization *in vivo* [94]. Furthermore MSC-derived exosomes modulate OC activity. Exosomes from cyclic mechanical stretch-treated BMSCs inhibit osteoclastogenesis by suppressing the NF-κB signaling pathway specifically through the inhibition of IκBα degradation reduced phosphorylation of IκBα, IKKα/β and p65 and prevention of p65 nuclear translocation thereby attenuating the expression of osteoclastogenesis-related genes thereby enhancing the bone density in a mouse model of disuse OP [95]. BMSC-derived exosomes promote M2 macrophage polarization through TRIM25-mediated ubiquitination of TREM1, enhancing osteogenic differentiation and alleviating osteoporosis. Similarly MSC-EVs enriched with miR-29a regulate angiogenesis by targeting VASH-1 while osteogenic MSC-EVs carry key proteins such as WNT5A, WNT5B, TGFB1, NOTCH2 and JAG1 along with osteogenic markers like SPP1, BSP and BGLAP driving bone regeneration. In summary MSC-derived exosomes orchestrate bone regeneration by simultaneously romoting osteogenesis angiogenesis immunomodulation and suppression of osteoclast activity through the concerted delivery of osteogenic proteins regulatory miRNAs and signaling modulators that reprogram multiple cellular targets within the bone microenvironment [96,97,178].

### Osteoblast-derived exosomes

OB-derived exosomes have are key mediators of bone regeneration, facilitating intercellular communication essential for bone homeostasis and repair [98]. These nanoscale, membrane-bound vesicles modulate the bone microenvironment, promote osteogenic differentiation, and enhance the recruitment and proliferation of MSCs at injury sites. OB-derived exosomes deliver osteoinductive factors such as proteins like BMP-9, OPG, EIF-2, and microRNAs (miRNAs) such as miR-30d-5p, miR-135B, and miR-677-3p, which are essential for osteogenesis [77]. They also inhibit OC formation through the miR-503-3p/Hpse axis, thereby preventing excessive bone resorption [99]. Although OB transplantation demonstrates the strongest regenerative effects, exosome-based treatments also enhance bone volume and trabecular architecture, particularly in the vertebral spine [100]. These findings position OB-derived exosomes as promising candidates for bone repair, and a novel strategy for enhancing bone regeneration and treating various bone-related disorders.

### Osteoclast-derived exosomes

OC-derived exosomes are key regulators of bone remodeling, facilitating intercellular communication within the bone microenvironment. These exosomes influence osteogenesis and bone resorption by delivering bioactive molecules to OBs and osteocytes, highlighting their potential for bone regenerative therapies [101,102]. A recent study identified miR-106a-5p-enriched OC-exosomes as key modulators of osteogenic differentiation, promoting bone regeneration in a mouse calvarial defect model by inhibiting *Fam134a* and upregulating osteogenic markers such as *Alp*, *Sp7*, *Col1a1*, and *Runx2* [103]. Additionally, inflammatory OC-derived exosomes deliver lncRNA LIOCE to OBs via ephrinA2/EphA2 signaling, stabilizing Osterix and enhancing OB activity. This mechanism increases bone volume and reduces bone porosity in *in vivo* models of osteolysis and femoral defects [104]. There have also been reports where Icariin-treated osteoclast-derived exosomes (ICA-OC-Exos) markedly enhanced OB activity and bone repair in infected bone nonunion models by increasing OB ALP and OCN expression while downregulating FGF23 levels. Similarly, OC small extracellular vesicles (OC-sEVs) were found to be highly enriched in miR-106a-5p, which suppresses Fam134a and significantly enhances osteogenic mineralization of BMSCs, ultimately promoting bone regeneration in a calvarial defect model [105,106]. This indicates that both MSC- and OC-derived exosomes synergistically drive bone regeneration by delivering osteogenic proteins, regulatory miRNAs, and signaling modulators that enhance OB activity, stimulate angiogenesis, reprogram immune responses, and suppress inhibitory pathways within the bone microenvironment.

### Osteocyte-derived exosomes

**Fig. 1:**
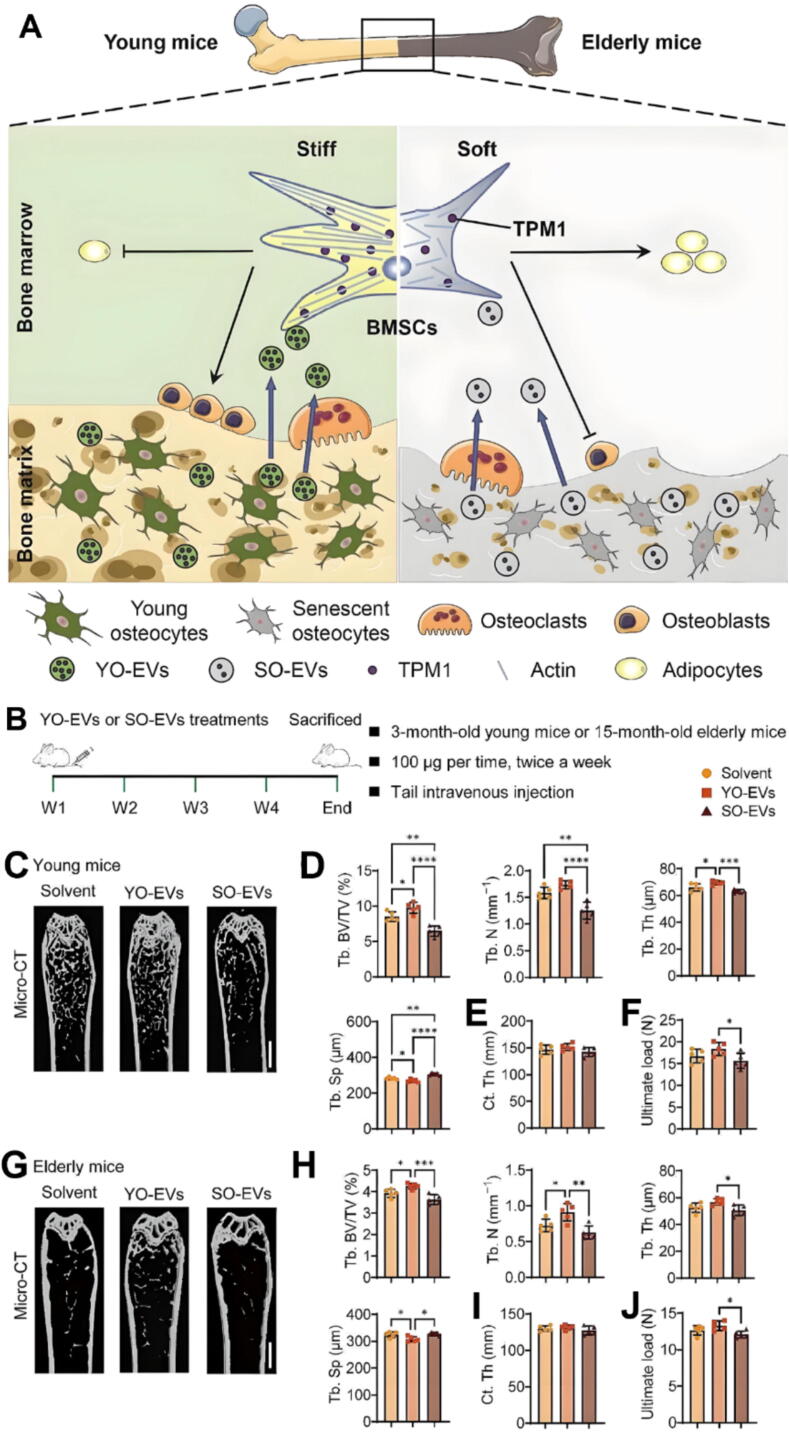
Therapeutic effects of YO-EVs on bone regeneration in young and elderly mice. (A) Schematic showing how YO-EVs promote BMSC osteogenesis. In young mice, YO-EVs, released from the bone matrix, deliver TPM1 into the bone marrow, stimulating osteogenesis by regulating F-actin polymerization. In elderly mice, reduced TPM1 delivery by SO-EVs disrupts the bone-fat balance, leading to OP. Administration of YO-EVs enhances bone regeneration in both young and elderly mice compared to that of SO-EVs and solvent controls. (B) Experimental protocol: 3-month-old (young) and 15-month-old (elderly) mice were administered tail vein injections of YO-EVs or SO-EVs (100 μg per injection, twice weekly) for 4 weeks, followed by euthanasia for analysis. (C–F) Representative micro-CT images and quantitative analysis of Tb. BV/TV, Tb. N, Tb. Sp, Ct. Th, and ultimate load in young mice treated with solvent, YO-EVs, or SO-EVs. YO-EV treatment significantly improved bone microarchitecture and mechanical strength compared with solvent or SO-EV treatments. (G–J) Micro-CT imaging and quantification of bone parameters in elderly mice treated with solvent, YO-EVs, or SO-EVs. YO-EV treatment significantly enhanced bone mass, trabecular structure, and mechanical properties in elderly mice, while SO-EV treatment promoted adverse effects, including increased adipogenesis. Figure A–I are distributed under the terms of the Creative Commons Attribution 4.0 International License. For details, see http://creativecommons.org/licenses/by/4.0/. YO-EVs, young-osteocyte-derived extracellular vesicles; TPM1, tropomyosin-1; SO-EVs, senescent-osteocyte-derived extracellular vesicles; OP, osteoporosis; Tb. BV/TV, trabecular bone volume fraction; Tb. N, trabecular number; Tb. Sp, trabecular separation; Ct. Th, cortical thickness.

Osteocytes, embedded within the bone matrix, are central regulators of bone homeostasis, coordinating bone remodeling through communication with OBs and OCs [107,108]. They release exosomes that mediate this interaction. Mechanically stimulated osteocyte-derived extracellular vesicles (MA-EVs), generated under fluid shear stress, were found to significantly promote the recruitment and osteogenic differentiation of human bone marrow stem cells (hMSCs), thereby supporting bone regeneration. Proteomic analysis revealed that these MA-EVs were enriched with proteins such as annexin A5 and histone H4, which upregulated osteogenic genes (COX2, OCN, OPN, RUNX2, OSX) and enhanced alkaline phosphatase activity, highlighting a novel osteocyte-mediated mechanosignaling mechanism for cell-free bone regeneration therapies [109]. Similarly, research on young osteocyte-derived EVs (YO-EVs) highlights their potential in combating age-related bone loss by promoting osteogenesis through increased matrix stiffness by F-actin polymerization and activation of osteogenic genes such as *RUNX2*. In contrast, senescent osteocyte-derived EVs (SO-EVs) promote adipogenesis, highlighting the influence of age-related changes on EV function (Fig. 1) [110]. Additionally, a study on mechanically stimulated osteocytes reports that their exosomes regulate OB and OC activity by delivering key signaling molecules such as RANKL, underscoring their role in maintaining the balance between bone formation and resorption [111]. Furthermore, fluid shear stress in 3D-cultured MLO-Y4 osteocytes markedly upregulated the pyrophosphate transporter Ank, and inhibiting Ank (via probenecid) significantly suppressed mechanically exosome production an effect that was rescued by exogenous pyrophosphate [112]. Additionally, under mechanical loading, osteocyte-derived exosomes enriched in miR-23b-3p target OTUD4 in chondrocytes, inhibiting mitophagy and skewing metabolism toward catabolism thereby disrupting cartilage homeostasis [113]. Thus, osteocyte-derived exosomes act as mechanoresponsive regulators that translate fluid shear stress and age-related changes into molecular signals such as annexin A5, histone H4, RANKL, and miR-23b-3p that directly modulate OB and OC activity, drive hMSC osteogenesis, and alter chondrocyte metabolism, thereby orchestrating bone regeneration and degeneration in a context-dependent manner.

### Macrophage-derived exosomes

Macrophages regulate bone regeneration by modulating inflammation, tissue remodeling, and repair via polarization into pro-inflammatory (M1) or anti-inflammatory (M2) states. Their exosomes facilitate communication with OBs, OCs, and BMSCs, influencing bone homeostasis [[114], [115], [116], [117], [118], [119], [120]]. Exosomes from M0- and M2-derived macrophages promote bone formation, while M1-derived exosomes inhibit it by delivering miR-155, which downregulates osteogenic genes such as *BMP2*, *BMP9*, and *RUNX2*. In contrast, M2 exosomes enriched with miR-378a enhance osteogenesis by upregulating osteoinductive genes [121]. Pulsed electromagnetic field stimulation shifts macrophages toward the M2 phenotype, reducing inflammation and producing exosomes that suppress osteoclastogenesis by downregulating CTSK, NFATC1, TRAP, miR-7, and miR-1897 [122]. M2-derived exosomes also inhibit RANKL-induced OC differentiation by downregulating CSF2 and inactivating TNF-α signaling, an effect enhanced by CSF2 knockdown or TNF-α inhibition [123]. Additionally, exosomes from M1 and M2 macrophages promote osteogenic differentiation of BMSCs, with M1-derived exosomes dominating the early inflammatory phase via miR-21a-5p, which upregulates markers such as *ALP*, *BMP-2*, *OPN*, *OCN*, and *RUNX2* [124]. Biomimetic periosteum constructs loaded with M2 macrophage–derived exosomes activate the Rap1/PI3K/AKT signaling pathway and enhance VEGF secretion, facilitating BMSC recruitment, angiogenesis, and osteogenic differentiation in early-stage bone defects [125]. In a preclinical model of osteonecrosis of the femoral head, M2-Exos enriched with miR-93-5p reduced TGF-β and PTEN expression while upregulating VEGFA and Akt, thereby improving endothelial function and vascular-immune crosstalk to support bone repair [126]. Therefore, macrophage-derived exosomes specifically mediate bone regeneration by delivering inhibitory miR-155 from M1 exosomes to suppress BMP2/9 and RUNX2-driven osteogenesis during early inflammation, while M2 exosomes enriched with miR-378a, miR-93-5p, and osteoinductive proteins activate Rap1/PI3K/AKT and VEGF signaling, suppress RANKL/TNF-α–induced osteoclastogenesis, and enhance BMSC recruitment, angiogenesis, and osteogenic differentiation, thus coordinating the transition from inflammatory resolution to tissue repair.

### iPSC-derived exosomes

iPSC-derived exosomes are promising candidates in regenerative medicine and therapeutic innovation due to their diverse bioactive cargo and ability to modulate cellular functions [127]. iPSCs, reprogrammed from somatic cells, differentiate into diverse cell types, and their exosomes exhibit this versatility. These vesicles carry noncoding RNAs, including miRNAs and long noncoding RNAs (lncRNAs), enabling them to influence target cells and alter pathological processes [128]. iPSC-derived exosomes demonstrate therapeutic potential across various diseases, including cardiovascular, neurodegenerative, and musculoskeletal disorders, by promoting tissue repair, modulating immunity, and reducing inflammation [[129], [130], [131], [132], [133]]. In bone regeneration, exosomes derived from human iPSC-derived mesenchymal stem cells (hiPSC-MSC-Exos) show potential in treating osteoporotic bone defects by enhancing osteogenic differentiation by upregulating the protein levels of RUNX-2, COL1, ALP, OPN, and OCN and bone regeneration in ovariectomized rat models [81]. Additionally, bioinspired nanovesicles (BNVs) derived from human iPSC-derived endothelial cells cultured under hypoxic conditions, enriched with CXCR4 and miR-1246, have been shown to reprogram bone marrow endothelial cells (BMECs). This reprogramming shifts their secretory profile toward increased production of VEGF, PDGFA, PDGFB, IL-10, TGF-β1, TGF-β2, and PGE2, which collectively promotes osteogenesis, suppresses adipogenesis, and supports M2 macrophage polarization, highlighting the therapeutic potential of BNVs in OP treatment. [134]. Moreover, iPSC-derived exosomes effectively treat osteonecrosis of the femoral head by enhancing angiogenesis through the activation of PI3K/Akt signaling pathway and preventing bone degeneration [135]. In summary, iPSC-derived exosomes contribute to bone regeneration by delivering osteoinductive cargo (RUNX2, COL1, ALP, OPN, OCN) that enhances osteogenic differentiation, reprogramming BMECs via CXCR4/miR-1246 to boost VEGF, PDGF, IL-10, and TGF-β secretion for angiogenesis and M2 polarization, and activating PI3K/Akt signaling to suppress adipogenesis and prevent bone degeneration, thereby integrating osteogenesis, vascularization, and immune modulation into a unified regenerative mechanism. Table 2 shows a summary of all the exosomes derived from various cellular sources.

**Table 2:** **Summary of therapeutic applications of different exosome types in bone regeneration and OP treatment.** This table presents various exosome sources including those derived from MSCs, OBs, OCs, osteocytes, macrophages, and iPSCs along with their respective administration dosages in *in vivo* and *in vitro* settings. The observed therapeutic outcomes are summarized, including osteogenic differentiation, angiogenesis, inhibition of OC activity, and bone mass enhancement. Notably, certain engineered or stimulated exosomes (e.g., cyclic stretch-treated BMSC-Exos, PEMF-stimulated M2 macrophage exosomes, and mechanically activated osteocyte-derived exosomes) demonstrate enhanced regenerative capacity through specific signaling pathways or miRNA/lncRNA cargo.

**Exosome type & source**		**Dosage/Administration**		**Observed therapeutic outcomes**	**Refs.**
		***In vivo***	***In vitro***		
MSC-derived exosomes	BMSC-derived exosomes	5 μg/ml	100 μg/50 μl	Increased OB proliferation and differentiation; enhanced new bone formation	[91]
	DPSC-derived exosomes + collagen sponge scaffold	0.0615 μg/μl	Not applicable	Enhanced bone volume and density	[92]
	Osteo-induced human MSC-derived exosomes	50 μg	10 μg/ml	Upregulated osteogenic gene expression; improved OB differentiation and mineralization	[93]
	Gingival MSC-derived exosomes	100 μl at a concentration of 1 μg/μl	50 μg/ml	Improved angiogenesis and osteogenesis; enhanced bone healing	[94]
	Cyclic stretch-treated BMSC-derived exosomes	5 mg/kg	25 μg/ml	Inhibited OC formation and function; increased bone mass and trabecular thickness	[95]
OB-derived exosomes	OB-derived exosomes	Not applicable	100 µg/mL	Inhibited OC formation via miR-503-3p targeting Hpse; downregulated OC marker genes	[77]
	OB-derived exosomes	Not applicable	100 µg	Improved bone volume, trabecular number, and architecture in lumbar vertebrae; less effective than OB transplantation, but significantly better than untreated OVX controls	[99]
OC-derived exosomes	OC-derived small exosomes enriched in miR-106a–5p	50 μg	1x10^9^ particles/ml	Inhibited Fam134a; upregulated Alp, Sp7, Col1a1, Runx2; enhanced bone regeneration	[103]
	Inflammatory OC (iOCL)-derived exosomes carrying lncRNA LIOCE	50 μg	Not specified	increased BV/TV and trabecular number; reduced bone porosity	[104]
Osteocyte-derived exosomes	Mechanically activated osteocyte-derived exosomes released under fluid shear stress	Not applicable	1 μg/well (24 well plate)	Exosomes significantly enhance hMSC recruitment and osteogenic differentiation	[109]
	Young osteocyte-derived EVs (YO–EVs) vs. senescent (SO–EVs)	100 μg	50 μg/ml	YO–EVs (enriched with tropomyosin–1) significantly enhance ALP activity, mineralization, and osteogenic gene expression in BMSCs and improve bone mass/strength *in vivo*; SO–EVs promote adipogenesis and reduce osteogenesis	[110]
	Exosomes from mechanically stimulated osteocytes delivering RANKL to OB and OC	Not applicable	Not specified	Osteocyte-derived exosomes regulate OB and OC functions	[111]
Macrophage −derived exosomes	M0, M1, M2 macrophage exosomes	8x10^9^	Not applicable	M0 & M2 exosomes promoted bone repair; M1 exosomes inhibited regeneration by suppressing BMP2, BMP9, RUNX2 via miR–155	[121]
	M2 macrophage exosomes obtained post- Pulsed Electromagnetic Field (PEMF) stimulation	Not applicable	10^6^/ml	PEMF shifted macrophages toward M2 phenotype; M2-exosomes more effectively reduced osteoclast activity and downregulated CTSK, NFATC1, TRAP, miR–7, miR–1897	[122]
	M2 macrophage exosomes (from IL to 4-polarized RAW264.7 cells)	Not applicable	50 μg/ml	M2-exosomes inhibited osteoclastogenesis by downregulating CSF2 and inactivating TNF–α signaling; CSF2 knockdown further enhanced this effect	[123]
	M1 macrophage exosomes enriched with miR–21a–5p		1 μg/ml	M1-derived exosomes (via miR–21a–5p) promoted early-phase osteogenesis: ALP, BMP2, OPN, OCN, RUNX2 expression upregulated	[124]
iPSC-derived exosomes	hiPSC-MSC-exosomes loaded on β–TCP scaffolds	200 μg	200 μg/ml	Dose-dependent enhancement of rBMSC-OVX proliferation, ALP activity, osteogenic gene expression; *in vivo*, dramatic stimulation of bone regeneration and angiogenesis	[81]
	Bioinspired, bone marrow endothelial cell–targeting nanovesicles (iPSC-derived)	1x10^10^ particles/50ul	1x10^9^	Converted skeletal endothelium-associated secretory phenotype, promoted BMSC osteogenesis, and ameliorated pro-inflammatory microenvironment	[134]
	iPSC-MSC-Exos (intravenous injection)	1x10^10^ particles/ml and 1x10^11^ particles/ml	Not applicable	Prevented bone loss in the femoral head and increased microvessel density via enhanced angiogenesis; tied to PI3K/Akt activation in endothelial cells	[135]

Beyond molecular engineering, hybrid platforms combining exosomes with advanced biomaterials and physical activation strategies are emerging as transformative approaches for bone repair. Recent biomaterials and exosome-engineered platforms are offering precise, context-adaptive solutions for bone repair in diabetic and neurogenic conditions. For instance, smart hydrogel scaffolds loaded with HA/MgO nanocrystals not only scavenge excess reactive oxygen species but also foster M2 macrophage polarization and upregulate osteogenic markers like Runx2 and OCN within diabetic bone defects [136]. In a complementary strategy, titanium implants modified via mussel-inspired adhesive and bioorthogonal click chemistry enable stable tethering of pre-osteogenic MSC-derived exosomes, which shift macrophage polarization toward a pro-regenerative phenotype and enhance expression of osteogenic genes in *peri*-implant tissues in diabetic rats [137]. Another cutting-edge approach employs acousto-electric fiber networks that, under ultrasound activation, stimulate Schwann cell–derived exosome release, leading to activation of Wnt and PI3K/Akt signaling pathways and resulting in neural-enhanced bone regeneration [138]. Together, these studies illustrate how bioactive scaffolds, exosome-functionalized implants, and responsive acousto-electric systems can be synergistically integrated to offer targeted, microenvironment-aware exosome-based therapies for complex bone healing scenarios.

## Translating bone regeneration to Osteoporosis: Exosomes as potential therapeutic agents

### Current knowledge gap

Translating bone regeneration strategies to OP therapy remains challenging due to fundamental differences in disease mechanisms. Bone regeneration focuses on activating OBs for new bone formation, while OP is characterized by excessive OC activity, impaired OB function, and disrupted bone remodeling [9,139,140]. Exosomes derived from regenerative models may not fully address these imbalances, particularly given the age-related decline in osteogenic capacity and reduced responsiveness of bone-resident cells such as OBs and BMSCs in OP [141,142]. This disparity underscores the need for OP-specific investigations rather than direct extrapolation from bone healing studies.

The osteoporotic microenvironment introduces additional complexity for exosome-based interventions. Factors such as altered systemic signaling, diminished intercellular communication, and comorbidities like diabetes or cardiovascular disease may influence exosome secretion, bioactive cargo, and therapeutic potential [[143], [144], [145]]. For example, BMSCs in osteoporotic conditions have been shown to release exosomes with modified molecular profiles compared to those from healthy bone, reducing osteogenic signaling and sometimes favoring adipogenic differentiation [146]. These variations highlight the importance of understanding disease-specific alterations in exosome biology to optimize their clinical application for OP.

Another significant challenge lies in the lack of standardized protocols for exosome isolation, characterization, and delivery, which limits reproducibility and comparability across studies [28,147]. Despite promising preclinical data, no consensus exists on optimal methods for ensuring purity, stability, and targeted delivery of therapeutic exosomes. Future strategies should aim to engineer exosomes for OP-specific purposes, such as surface modification for OC targeting, loading osteogenic miRNAs, or incorporating bone-homing peptides [148]. Addressing these challenges could establish exosome-based therapy as a precise, minimally invasive, and highly adaptable alternative to conventional pharmacological treatments for OP.

### Potential mechanisms in osteoporosis

Current treatments for OP primarily involve anti-resorptive or anabolic drugs; however, their effectiveness is often limited by side effects and declining efficacy over time [[149], [150], [151], [152]]. Consequently, exosomes have gained increasing interest as a novel therapeutic avenue for OP [153]. Since direct studies on OP-specific exosome applications remain limited, findings from bone regeneration models provide valuable insights into their potential mechanisms and therapeutic utility.

One of the primary mechanisms through which exosomes act in OP through promoting osteogenesis and enhancing OB function, which is often impaired by aging, estrogen deficiency, and inflammatory cytokines [154]. For instance, MSC-derived exosomes are potent inducers of OB differentiation, driven by their rich cargo of osteogenesis-related miRNAs such as miR-335, miR-214-3p, and miR-26a. These miRNAs upregulate key osteogenic transcription factors, including RUNX2, OPN, Osterix (SP7), and Collagen Type I (COL1A1), thereby stimulating OB proliferation and matrix mineralization [[155], [156], [157]]. Recent advances in exosome preconditioning show that hypoxic preconditioning of MSCs enhances their osteogenic potential by upregulating hypoxia-inducible factor 1-alpha (HIF-1α), increasing the secretion of pro-osteogenic exosomes [158]. Another promising approach is the genetic modification of exosome-producing cells to overexpress osteogenic transcription factors such as RUNX2 or BMP-2, enhancing their ability to stimulate bone formation [159,160]. Moreover, studies have explored the combination of exosomes with bioactive scaffolds by incorporating engineered exosomes loaded with osteogenic factors into 3D-printed or hydrogel-based delivery systems, creating sustained-release platforms for bone regeneration [161,162]. These approaches have enhanced bone healing in preclinical models and show strong potential for translating exosome-based therapies into clinical OP treatment.

Beyond stimulating osteogenesis, exosomes regulate osteoclastogenesis and prevent excessive bone resorption, a key feature of OP. OC, derived from hematopoietic precursors, is activated through receptor activators of nuclear factor kappa-B ligand (RANKL) signaling, which stimulates their differentiation and resorptive activity [163,164]. Studies have demonstrated that exosomes derived from MSCs carry miR-503, which reduces bone resorption by inhibiting OC activity, while exosomes from HUVECs deliver miR-21-5p, thereby enhancing bone formation. [165,166]. Another innovative approach involves engineering exosomes with OC-targeting peptides, such as D-aspartic acid motifs, which selectively bind hydroxyapatite (bone resorbed area) and deliver inhibitory cargo to suppress their resorptive activity [167].

In addition to their direct effects on OBs and OCs, exosomes modulate the inflammatory microenvironment, a critical factor in OP pathogenesis. Chronic low-grade inflammation, particularly in postmenopausal OP, is associated with increased levels of pro-inflammatory cytokines such as tumor necrosis factor-alpha (TNF-α), interleukin-6 (IL-6), and interleukin-1 beta (IL-1β), which promote bone resorption and inhibit osteogenesis [168]. Exosomes from various cell sources exhibit potent immunomodulatory properties by delivering anti-inflammatory miRNAs, including miR-146a and miR-21, which downregulate NF-κB signaling and suppress the production of OC-activating cytokines [169,170]. Furthermore, BMSC-derived exosomes promote the polarization of M2 anti-inflammatory macrophages, which secrete factors that enhance osteogenic differentiation and reduce bone resorption [171]. Additionally, regulatory T cell (Treg)-derived exosomes modulate immune-osteogenic crosstalk, promoting the differentiation of osteogenic progenitor cells and reducing inflammatory bone resorption [172]. These findings underscore the potential of exosomes as osteoanabolic agents and immunotherapeutic tools for OP treatment.

Incorporating bioengineering approaches has further enhanced the therapeutic potential of exosomes for OP. One of the most promising innovations is the development of hybrid exosomes, created by fusing natural exosomes with synthetic nanoparticles to enhance their stability, targeting efficiency, and controlled release [173]. These hybrid vesicles can be functionalized with bone-targeting ligands, such as alendronate or hydroxyapatite-binding peptides, ensuring selective accumulation in osteoporotic bone tissue [174]. Another breakthrough is the use of stimuli-responsive exosome delivery systems, where engineered exosomes are encapsulated in hydrogels or polymeric carriers that degrade in response to pH changes in osteoporotic bone lesions or enzymatic activity associated with bone remodeling [175,176]. This approach enables sustained, localized exosome release, maximizing their therapeutic effects while minimizing off-target interactions.

In conclusion, although research on exosome-based therapies for OP remains in its early stages, evidence from bone regeneration models highlights their strong potential to target the multifaceted mechanisms underlying OP. By promoting osteogenesis, inhibiting osteoclastogenesis, and modulating inflammation, exosomes represent a promising alternative to conventional OP treatments.

### Osteoporosis subtype-specific considerations for exosome therapy

Postmenopausal osteoporosis (PMOP) is driven by abrupt estrogen deficiency, amplifying RANKL-mediated osteoclastogenesis and inflammatory signaling. In contrast, senile osteoporosis (SOP) reflects an aged bone milieu characterized by stem-cell exhaustion, osteocyte/OB senescence, oxidative stress, and low-grade inflammation, collectively impairing bone formation and quality. These pathophysiological distinctions imply different therapeutic priorities: anti-resorptive, immunomodulatory exosomal cargo is most relevant for PMOP, whereas SOP requires rejuvenation-oriented, pro-osteogenic/angiogenic and anti-senescence cargo [28,[177], [178], [179]].

Preclinical evidence supports these subtype-specific considerations. In OVX rodents (PMOP analogues), endothelial cell–derived exosomes (EC-Exos) reduced osteoclast activity and protected bone, with miR-155 identified as a key anti-resorptive cargo consistent with estrogen-withdrawal biology [180]. By contrast, SOP-focused studies often use naturally aged models or compare young versus aged donor exosomes. Young plasma-derived exosomes consistently enhanced osteogenesis and reduced resorption, outperforming aged exosomes across several endpoints. Similarly, serum/plasma exosomes from young donors restored osteogenic potential and ameliorated bone loss in aged rodents, underscoring a rejuvenation mechanism specific to SOP [181].

Mechanistic insights further reinforce these subtype distinctions. For PMOP, down-modulation of NF-κB/RANKL signaling and osteoclastogenesis (e.g., via miR-155 cargo from EC-Exos) is most beneficial, whereas in SOP the emphasis lies on counteracting cellular senescence/oxidative stress and promoting osteoblastogenesis and angiogenesis. Reviews on bone aging and EV biology highlight that EV biogenesis and cargo composition shift unfavorably with age, suggesting that young-source or engineered EVs are likely to be more effective in SOP [178,189].

From a translational perspective, circulating exosome profiles already distinguish osteoporotic postmenopausal women from osteopenic or healthy controls, supporting exosome-based stratification in PMOP where inflammatory and resorptive axes dominate [146]. PMOP may particularly benefit from bone-targeted exosomes that concentrate anti-resorptive payloads at trabecular bone surfaces (e.g., acidic aspartate–rich (DSS)_6_ ligands or apoptotic exosomes engineered with (DSS)_6_ and osteoanabolic cargos). For SOP, strategies that enhance cargo quality through young-source exosomes, preconditioning, or engineering and approaches that enable osteocyte-directed delivery are especially relevant given the central role of osteocyte senescence [182,183]. Therefore, exosome-based therapy for OP must be subtype-tailored: in PMOP, endothelial exosome–derived miR-155 and other anti-resorptive cargos that suppress NF-κB/RANKL-driven osteoclastogenesis address estrogen-withdrawal–induced bone loss, whereas in SOP, young-source or engineered exosomes enriched with pro-osteogenic, angiogenic, and anti-senescence factors restore OB function, counter oxidative stress, and rejuvenate osteocytes, thereby directly targeting the aging-associated decline in bone formation and quality.

### Potential for targeted therapy and controlled release

A key advantage of using exosomes in OP treatment is their capacity for engineering to enable targeted delivery to specific tissues. In OP, directing therapeutic agents specifically to bone tissue can significantly enhance efficacy by concentrating bioactive molecules at the site of action [47,184].

Recent advances in exosome engineering have enabled the modification of surface proteins to facilitate specific binding to bone tissue or bone-resorbing cells, such as OCs. For instance, incorporating bone-targeting peptides, such as those binding to hydroxyapatite or integrins expressed on OCs, can enhance the accumulation of exosomes in osteoporotic bone, thereby improving the localized delivery of their therapeutic cargo [185,186]. This targeted delivery approach may increase the efficacy of exosome-based therapies while reducing potential off-target effects.

**Fig. 2:**
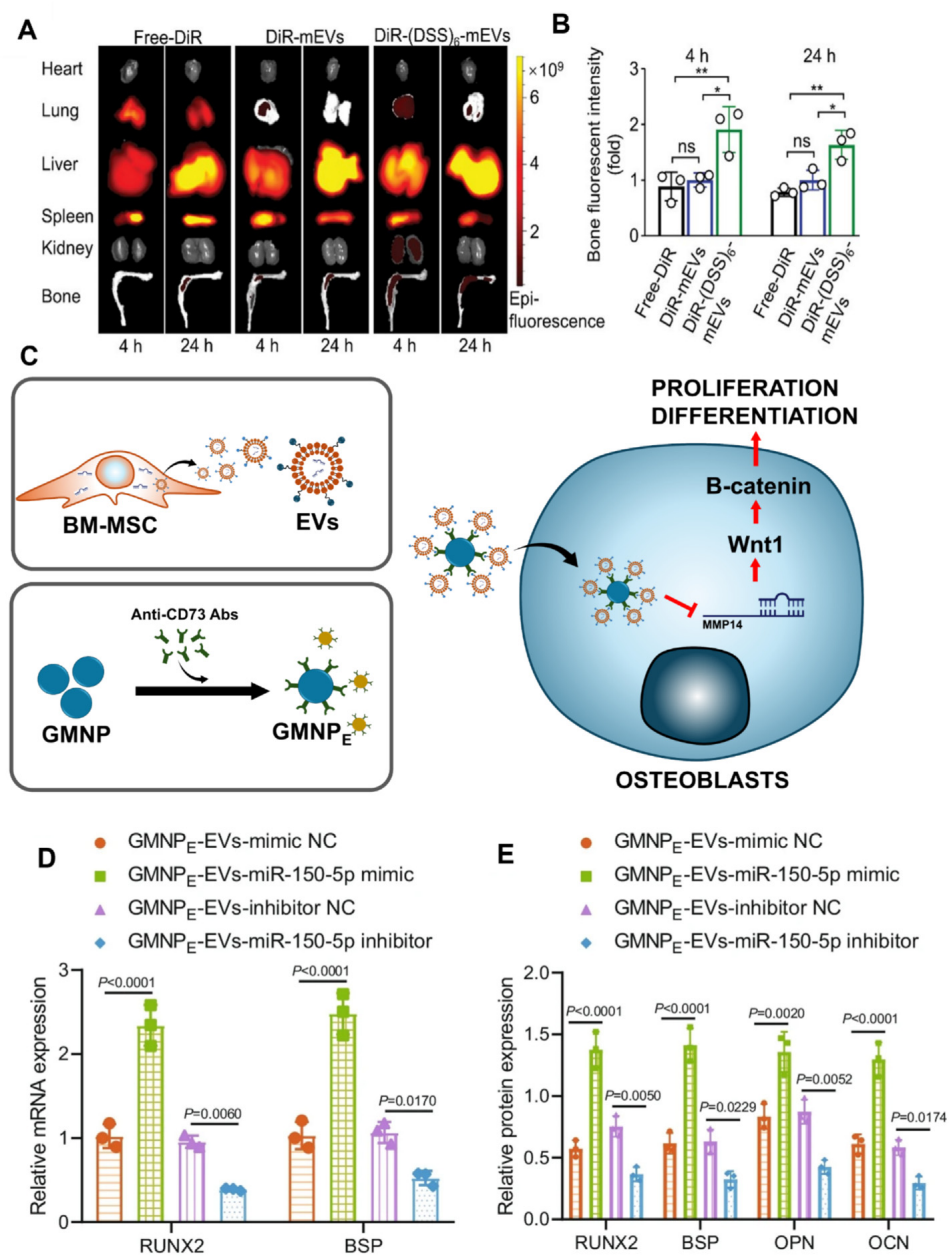
*In vivo* distribution of (DSS)_6_-mEVs assessed via IVIS imaging: DiR-labeled (DSS)_6_-mEVs and mEVs were intravenously injected into mice, with free-DiR serving as a control. Major organs, tibia, and femur were collected for imaging at 4-h and 24-h post-injection. (A) (DSS)_6_-mEVs-treated mice exhibited a stronger fluorescent signal in the bone than those in the mEVs-treated and free-DiR-treated groups. (B) Quantitative analysis revealed a 1.9-fold and 1.6-fold increase in fluorescence intensity for (DSS)_6_-mEVs at 4 and 24 h, respectively, compared to mEVs-treated mice. (C) A schematic representation illustrates how BMSC-EV-loaded GMNPs modulate the progression of DO. GMNPs encapsulating BMSC-derived EVs serve as carriers for miR-150-5p, delivering it to OBs, where it targets and suppresses MMP14 expression, leading to the activation of the Wnt/β-catenin signaling pathway. This pathway activation promotes the proliferation and maturation of OBs, thereby contributing to the delayed progression of DO. In the schematic, black upward arrows indicate the upregulation of gene expression, while downward arrows represent downregulation, highlighting the molecular changes induced by this therapeutic strategy. (D) Relative mRNA expression levels of RUNX2 and BSP analyzed via RT-qPCR revealed the upregulation of osteogenesis-related genes. (E) Immunoblotting analysis revealed elevated protein expression of RUNX2, BSP, OPN, and OCN in OBs. Figures C, D, and E are distributed under the terms of the Creative Commons Attribution 4.0 International License. For details, see http://creativecommons.org/licenses/by/4.0/. DO, distraction osteogenesis; GMNP, gold-coated magnetic nanoparticle; BMSC, bone marrow mesenchymal stem cell; EV, extracellular vesicle; OB, osteoblast; IVIS, *in vivo* imaging system; RT-qPCR, reverse transcription-quantitative polymerase chain reaction; RUNX2, runt-related transcription factor 2; BSP, bone sialoprotein; OPN, osteopontin; OCN, osteocalcin.

Recent advancements in engineered exosomes for OP treatment include the development of milk-derived exosomes conjugated with the bone-targeting peptide (Asp-Ser-Ser)_6_ [DSS_6_] via click chemistry. These exosomes, loaded with the SIRT1 agonist SRT2104 and MnB nanoparticles for MRI visualization, effectively promote osteogenesis and inhibit osteoclastogenesis. *In vivo* studies using ovariectomized mice show increased bone mineral density, higher trabecular volume (BV/TV), and stronger fluorescence signals at bone sites, confirming the bone-targeting efficacy of these engineered exosomes as a promising theranostic platform for OP treatment and monitoring (Fig. 2A–B) [187]. Additionally, a novel approach for treating diabetic OP (DO) utilized BMSC-derived exosomes loaded onto gold-coated magnetic nanoparticles (GMNPs) to deliver miR-150-5p. This delivery system enhanced osteogenesis by activating the Wnt/β-catenin signaling pathway and upregulating osteogenic markers, leading to improved OB proliferation and maturation in a diabetic rat model (Fig. 2C–E) [188]. In line with these findings, Table 3 provides a summary of different exosome sources and their contributions to bone repair.

**Table 3:** **Summary of therapeutic roles of exosome-derived miRNA cargo in bone regeneration and OP models.** The table presents different exosome sources, their cargo characterization or bioengineering strategy, reported dosage and administration *in vivo* and *in vitro*, as well as the corresponding therapeutic outcomes. MSC-derived exosomes are shown to carry osteogenic or immunomodulatory miRNAs (e.g., miR-26a, miR-335, miR-503-5p, miR-146a, miR-21, miR-150-5p), contributing to enhanced osteoblast differentiation, fracture healing, reduced osteoclastogenesis, and improved bone microarchitecture. Non-MSC exosomes, including those derived from HUVECs and Treg cells, also exert osteogenic and immunoregulatory effects through modulation of angiogenesis, YAP1 signaling, and macrophage polarization. Together, these findings highlight the multifaceted therapeutic potential of exosomes as modulators of bone formation, resorption, and immune balance in osteoporotic conditions.

**Exosomes types and characterization/strategy**		**Reported Dosage/ Administration**		**Observed therapeutics outcomes**	**Reference**
		***In vivo***	***In vitro***		
MSC-derived exosomes	miR-214-3p (downregulated in MSC-Exos upon mechanical loading)	Not specified	25 μg/ml	Mechanical loading decreased miR-214-3p in MSC-Exos, enhancing type H vessel formation, indirectly supporting osteogenesis and angiogenesis	[156]
	miR-335	100 μl	Not applicable	miR-335 in BMSC-EVs targets VapB, activates Wnt/β-catenin, enhances OB differentiation, and accelerates fracture healing	[158]
	miR-503-5p	8x10^9^ exosomes	1 μg/μL	↓ OC activity, ↓ bone resorption, ↑ bone mass	[164]
	miR-146a	1.6 mg/kg	1 μg/μl per well (well plate not specified)	↓ TNF-α, IL-6; ↓ NF-κB activity; ↓ osteoclastogenesis; ↑ bone microarchitecture	[169]
	miR-21	1.6 μg/μl	Not applicable	↓ RANKL, TNF-α, IL-1β; ↓ NF-κB signaling; ↑ bone density and trabecular structure	[170]
		20 mg	2 μg of exosome per 1x10^5^ cells	↑ M2 macrophages (CD206 + ); ↑ IL-10 secretion; ↑ osteoblast	[171]
	miR-150-5p	10 mg/kg	Not mentioned	↑ OB proliferation & maturation; ↑ bone formation; ↑ mineralization; ↓ diabetic bone loss progression	[188]
HUVEC-derived exosomes	miR-21-5p	200 μg	20 μg/ml	↑ Osteogenic markers (RUNX2, ALP, OPN), ↑ bone regeneration via YAP1 modulation	[166]
Treg cell-derived exosomes	Not applicable	150 μg/ml	10 μg/ml	↑ M2 polarization via STAT6; ↑ osteogenic gene expression (Runx2, ALP); ↓ inflammatory cytokines; ↑ fracture healing	[172]

### Clinical translation of exosome-based therapies for osteoporosis

Exosome-based therapies for OP are still in the early stages of development, their potential for clinical translation is significant. The low immunogenicity of exosomes, along with their ability to transport diverse bioactive molecules and facilitate targeted delivery, positions them as promising candidates for clinical application [148,189,190]. However, challenges such as large-scale production, regulatory approval, and long-term safety require resolution before widespread clinical use. In conclusion, exosomes demonstrate significant potential as a therapeutic approach for OP by modulating OB and OC activity, regulating the inflammatory microenvironment, and enabling targeted, controlled delivery of therapeutic agents.

### Challenges in exosome-based therapies for osteoporosis

Exosomes offer a promising therapeutic approach for OP due to their inherent roles in intercellular communication and tissue regeneration. However, clinical application faces several biological and technical challenges that must be addressed to realize their full therapeutic potential for OP. These challenges encompass heterogeneity in exosomal content, limited targeting specificity, insufficient stability, and difficulties related to their isolation, characterization, and delivery to exosomes to osteoporotic bone tissue. The following section provides a detailed discussion of these challenges.

### Biological challenges

#### Variability in exosome content

A major challenge in using exosomes for OP therapy is the inherent variability in their molecular cargo. Exosomes exhibit heterogeneity in their content which can significantly affect their therapeutic efficacy. Several factors influence exosome composition, including the cell of origin (e.g., OB, MSC, or macrophages), the production conditions, and the differentiation or activation state of the donor cells [191]. In OP, where precise modulation of bone formation and resorption is critical, variability in exosome content may lead to inconsistent or suboptimal therapeutic effects. Standardizing exosome composition using techniques such as electroporation, extrusion, and saponin-assisted loading is essential to ensure reproducibility across batches and overcome this challenge [192,193]. Furthermore, identifying the “ideal” exosome composition for effectively targeting OP remains a critical area of ongoing research.

#### Targeting specificity

Another key biological challenge in exosome-based therapy for OP is achieving specific targeting of osteoporotic bone tissue or key cell types involved in bone remodeling, such as OCs and OBs. While exosomes exhibit inherent tissue tropism, their natural targeting mechanisms are often suboptimal for bone tissue, particularly in OP, where the balance between OB and OC activity is disrupted.

Targeting specificity is influenced by several factors, such as the surface markers expressed on exosomes, the molecular composition of their cargo, and the receptor profiles of the recipient cells [194,195]. For example, MSC-derived exosomes demonstrate a natural tendency to home to sites of injury or inflammation; however, their targeting efficiency toward bone tissue remains inconsistent [196]. Additionally, OCs and OBs may lack sufficient expression of the required receptors for efficient exosome uptake, further complicating targeted delivery. To address this challenge, exosomes can be bioengineered by modifying their surfaces with bone-targeting ligands, peptides, antibodies that recognize receptors specific to bone tissue or those expressed on OCs or OBs [38,197]. However, optimizing these targeting strategies requires a deeper understanding of the molecular interactions between exosomes and their recipient cells.

#### Exosome stability

Exosome stability is a crucial determinant of the effectiveness of exosome-based therapies. Following isolation and purification, exosomes must be stored, transported, and delivered to the target tissue while preserving their bioactive cargo and functional integrity [[198], [199]]. However, their lipid bilayer structure makes them susceptible to degradation, and improper storage or handling can compromise cargo stability [200,201]. This instability poses a significant challenge to clinical application, as degradation may diminish therapeutic efficacy.

Several factors affect exosome stability, including temperature, pH, and exposure to enzymes or other environmental conditions that may degrade their bioactive cargo. Additionally, repeated freeze–thaw cycles during storage may cause structural damage, thereby compromising their functional integrity [202,203]. To address these challenges, strategies such as the use of cryoprotectants, surfactants, or polymeric coatings have been investigated to preserve exosome stability during storage and delivery [194,204,205]. However, further research is necessary to optimize these methods for long-term preservation and ensure exosomes retain their bioactivity throughout the therapeutic process.

### Technical limitations

#### Isolation of exosomes

The isolation and purification of exosomes are critical steps in preparing them for therapeutic applications. A major obstacle to clinical translation of exosome-based therapies lies in the lack of standardized isolation techniques. Current methods, including ultracentrifugation, precipitation, size-exclusion chromatography, immunoaffinity capture, and microfluidic platforms, yield exosome preparations that differ markedly in purity, recovery efficiency, and functional bioactivity [[206], [207], [208], [209]]. This methodological variability complicates cross-study comparisons and hinders reproducibility, as different protocols may enrich distinct extracellular vesicle subpopulations or co-isolate protein aggregates and lipoproteins [210]. Although the Minimal Information for Studies of Extracellular Vesicles (MISEV2018) guidelines from the International Society for Extracellular Vesicles (ISEV) provide broad recommendations for reporting and characterization, they do not establish disease- or application-specific protocols [211]. Importantly, for OP research, no consensus exists regarding optimal isolation strategies tailored to bone-related exosomes. From a translational perspective, the absence of Good Manufacturing Practice (GMP)-compliant workflows further restricts regulatory approval and clinical scalability [212]. Recent efforts such as tangential flow filtration (TFF) combined with chromatography have shown promise in improving yield, purity, and cost-efficiency while minimizing contamination from serum-derived exosomes [213]. However, systematic benchmarking studies and validation of quality-control metrics, such as particle size distribution, exosomal marker expression (CD63, CD81, TSG101), and contaminant profiling remain urgently needed to ensure reproducibility and standardization across laboratories. Additionally, the yield of exosomes from primary sources, such as human MSCs or OBs, remains limited, and scalable production strategies are still under active development [[214], [215], [216], [217]]. Achieving efficient isolation and purification of high-quality exosomes from clinical-grade sources remains a key challenge in advancing exosome-based therapies for OP.

#### Characterization of exosomes

Comprehensive characterization of exosomes is essential for ensuring their consistency, quality, and therapeutic functionality. However, current techniques remain insufficiently standardized, and no universally accepted method exists for assessing exosome identity, size, and molecular composition. Traditional methods, such as nanoparticle tracking analysis (NTA), dynamic light scattering (DLS), and electron microscopy, offer insights into size distribution and morphology but fall short of delivering a comprehensive assessment of the molecular cargo or functional properties of the vesicles. Furthermore, the presence of non-exosomal particles or contaminants, such as free proteins or lipoproteins, complicates the characterization process. High-resolution techniques, such as mass spectrometry and RNA sequencing, are increasingly employed to analyze the molecular composition of exosomes. However, these methods require extensive validation and remain costly and time-intensive, limiting their routine application in clinical and translational settings [191,[218], [219], [220], [221]]. Developing standardized protocols for exosome characterization is crucial to ensure the reproducibility and reliability of exosome-based therapeutic applications.

#### Delivery of exosomes to osteoporotic bone tissue

Delivering exosomes to osteoporotic bone tissue remains one of the most significant technical challenges in advancing exosome-based therapies for OP. Osteoporotic bone exhibits reduced mass, disrupted microarchitecture, and changes in the bone marrow microenvironment, all of which can impair the effective delivery of therapeutic agents. Additionally, the ability to selectively target exosomes to osteoporotic bone tissue or cells involved in bone remodeling is essential for maximizing their therapeutic efficacy [[222], [223], [224]].

Several strategies have been investigated to enhance exosome delivery to bone tissue, including surface modification with targeting peptides or antibodies, encapsulation within biomaterials, and incorporation into nanoparticles to enhance circulation time and protect the vesicles from degradation [38,196,225]. However, ensuring that exosomes effectively reach the bone tissue and are efficiently internalized by target cells, such as OBs or OCs, remains a significant challenge.

Although exosomes offer significant potential for treating OP, successfully translating exosome-based therapies into clinical practice requires overcoming several biological and technical challenges. Variability in exosome cargo, limited targeting specificity, instability, and challenges related to isolation, characterization, and delivery represent key barriers to optimizing exosome-based therapies for OP. However, ongoing research and technological advancements can potentially overcome these challenges, positioning exosomes as a promising and effective therapeutic strategy for OP management and bone regeneration. Table 4 summarizes the key challenges associated with exosome-based drug delivery for OP.

**Table 4:** **Innovations and future directions in exosome-based therapies for OP**. This table summarizes emerging strategies to optimize exosome-based treatments for OP. It categorizes current advancements into engineered exosomes for targeted delivery and improved stability, RNA-loaded exosomes for precise modulation of bone remodeling, and immunomodulatory approaches to control inflammation. Together, these strategies aim to overcome translational hurdles and enhance therapeutic efficacy in osteoporotic bone regeneration.

**Category**	**Challenge**	**Details**	**References**
**Biological Challenge**	Variability in exosome content	Exosome cargo varies with donor cell type, state, and culture conditions, influencing their therapeutic effect in OP	[[191], [192], [193]]
	Targeting specificity	Natural exosome targeting is limited by surface markers, cargo, and receptor expression	[38,195,196]
	Exosome stability	Exosomes degrade during storage and delivery; stability depends on temperature, pH, and freeze–thaw cycles	[[202], [203], [204], [205]]
**Technical Limitation**	Isolation of exosomes	Current methods (e.g., ultracentrifugation) are time-consuming, low-yield, and can damage vesicles	[[214], [215], [216], [217]]
	Characterization of exosomes	Techniques like NTA, DLS, and EM offer limited info. Lack of standardized, comprehensive characterization of size, identity, and cargo	[[218], [219], [220], [221]]
	Delivery to osteoporotic bone	OP alters the bone structure and microenvironment, complicating exosome delivery. Effective targeting and uptake by OBs or OCs is still difficult	[38,196,225]

## Innovations and future directions for exosome-based therapies in osteoporosis

Exosomes offer significant potential for treating OP by enhancing bone regeneration and modulating bone metabolism. However, translating exosome-based therapies from preclinical studies to clinical applications requires overcoming several challenges, particularly those related to targeting specificity, efficient cargo delivery, and precise tissue localization. To address these limitations, several innovative strategies have been developed and are continuously evolving. These strategies include engineering exosomes to enhance their therapeutic efficacy, combining exosomes with complementary treatment modalities, and leveraging RNA-based interventions and immunomodulatory mechanisms to optimize their effects. The following sections provide a detailed overview of these emerging innovations.

### Engineered exosomes

Engineered exosomes mark a significant advancement in exosome-based therapies, offering promising solutions to challenges related to targeting specificity, cargo delivery, and therapeutic functionality. While natural exosomes facilitate intercellular communication and support tissue regeneration, they may lack the precision needed to target osteoporotic bone or deliver therapeutic cargo effectively. To address these limitations, engineered exosomes can be tailored to enhance targeting accuracy, cargo loading, and therapeutic outcomes.

### Enhanced targeting of osteoporotic bone

Surface-conjugation of bone-targeting peptides to exosome membranes can markedly increase bone accumulation and improve bone microarchitecture in OVX models. Cui et al. developed a bone-targeted exosome (BT-Exo) platform by grafting a periostin-binding/osteoblast-targeting peptide onto iPSC-MSC-derived exosomes and loading them with siRNA (BT-Exo-siShn3). Compared with unmodified exosomes, BT-Exo-siShn3 showed stronger uptake by osteoblast lineage cells *in vitro* (DiR/DiI imaging and FCM/CLSM) and greater bone accumulation *in vivo*, and produced statistically significant improvements in bone parameters measured by micro-CT and histomorphometry (increased BV/TV and Tb.N, higher MAR, and reduced Tb.Sp and OC number), as well as reduced serum bone-resorption markers and increased osteogenic markers. The study explicitly links surface peptide targeting to enhanced osteoblast delivery, leading to a measurable rescue of OVX-induced bone loss, demonstrating that peptide surface functionalization can translate into quantifiable, biologically meaningful gains in bone mass and microarchitecture [35].

**Fig. 3:**
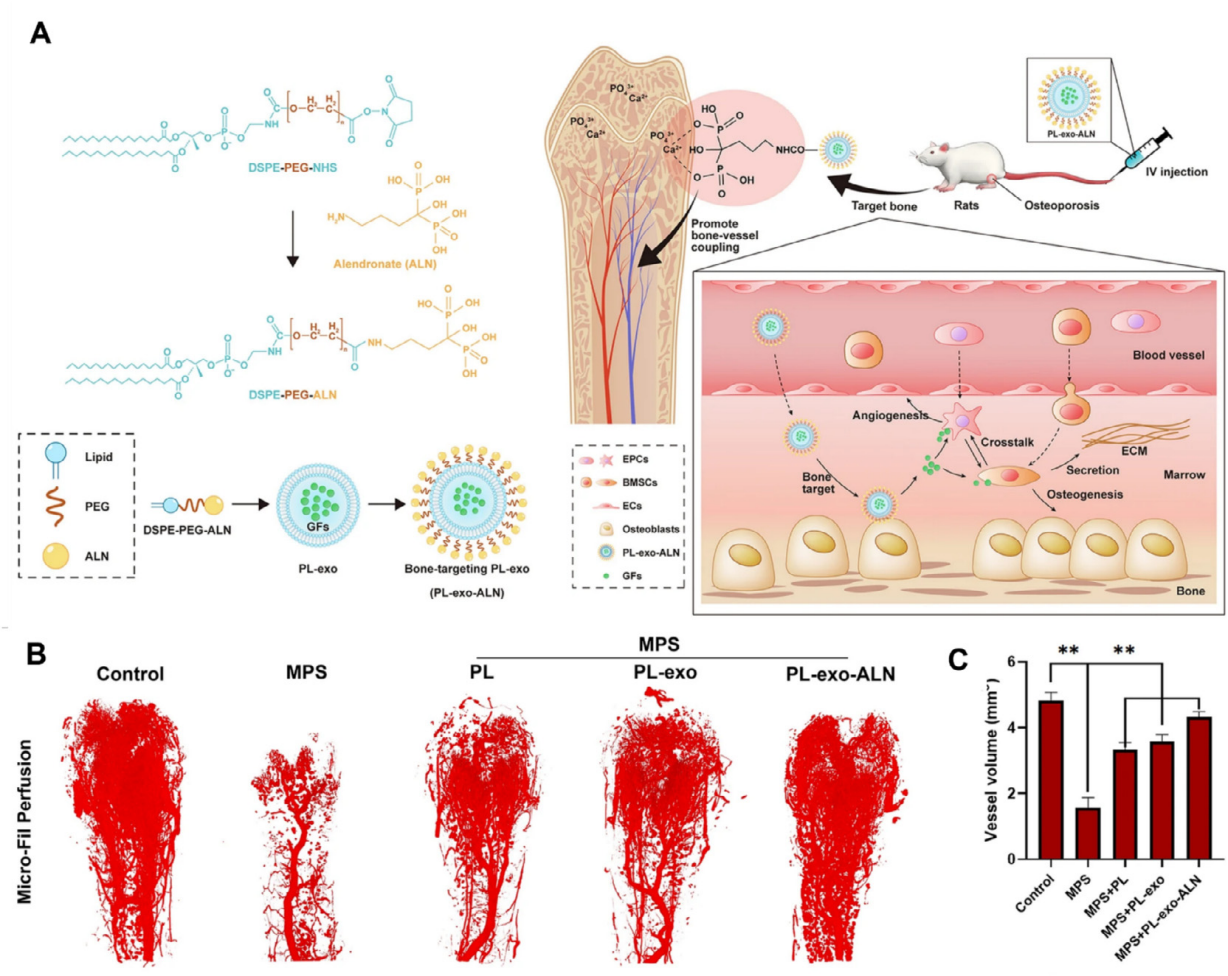
Bone-targeted exosome strategy and restoration of intraosseous vasculature in glucocorticoid-induced osteoporosis. (A) Schematic showing the functionalization of platelet-lysate-derived exosomes (PL-exo) with DSPE-PEG-alendronate (ALN) to enhance hydroxyapatite affinity and bone accumulation, thereby promoting osteogenesis and angiogenesis.

A different but complementary approach uses small-molecule bone anchors (bisphosphonates) inserted into exosome membranes to increase hydroxyapatite affinity and in-vivo bone enrichment, a strategy that produces clear, reported changes in binding and therapeutic outcomes. Zheng et al. modified platelet-lysate-derived exosomes with DSPE-PEG-alendronate (ALN) and quantified the change in hydroxyapatite (HAp) binding and biodistribution: QCM-D and HAp-particle binding assays showed the *in vitro* HAp binding fraction increased from ∼ 21 % (unmodified PL-exo) to ∼ 78 % after ALN functionalization, and IVIS biodistribution confirmed markedly higher bone marrow accumulation *in vivo*. PL-*exo*-ALN treatment significantly reversed glucocorticoid-induced bone loss by improving bone mineral density and trabecular architecture (BV/TV, Tb.N, Tb.Th, Tb.Sp) on micro-CT, enhancing osteogenic differentiation markers (RUNX2, Col-I, OCN), elevating ALP activity and calcium deposition, and reducing TRAP-positive OC surface. In parallel, Micro-Fil perfusion and 3D micro-CT vascular reconstruction showed restored intraosseous vessel volume, with higher VEGF expression and increased CD31^+^/EMCN^+^ type H vessels, demonstrating concurrent osteogenic and angiogenic recovery (Fig. 3) [226].

(B) Representative three-dimensional (3D) Micro-CT reconstructions of intraosseous vasculature in rat distal femurs following Micro-Fil perfusion and decalcification. Five groups are shown: Control (normal vasculature), MPS (marked vascular loss due to methylprednisolone-induced osteoporosis), PL (partial vascular restoration after platelet lysate treatment), PL-exo (greater vascular recovery due to exosome treatment), and PL-*exo*-ALN (maximal restoration, indicating synergistic benefit of ALN modification). PL-*exo*-ALN treatment produced dense and continuous vascular networks compared to the sparse and fragmented vasculature seen in the MPS group.

(C) Quantitative analysis of vessel volume from 3D reconstructions reveals significant differences among groups. MPS markedly reduced vessel volume compared to control. PL and PL-exo partially reversed this reduction, while PL-*exo*-ALN significantly restored vessel volume to near-normal levels, confirming superior angiogenic efficacy. Adapted from Zheng et al., distributed under the terms of the Creative Commons Attribution 4.0 International License (http://creativecommons.org/licenses/by/4.0/).

### Preconditioning of donor cells

Preconditioning involves exposing donor cells to specific environmental stimuli such as hypoxia, mechanical stress, inflammation, or pharmacological agents before collecting their exosomes. This strategy induces cellular responses that modify the composition of secreted exosomes, thereby enhancing their therapeutic potential. The primary advantage of preconditioning is its ability to enrich exosomes with targeted bioactive molecules, including proteins and non-coding RNAs (such as miRNAs), which are critical for modulating cellular behavior and promoting tissue regeneration [227].

For OP, a systemic skeletal disorder characterized by impaired bone remodeling and chronic inflammation, preconditioning donor cells can be strategically used to generate exosomes that specifically address these pathological features. This preconditioning process can be optimized to enhance the delivery of exosomes with strong anti-inflammatory, osteogenic, and bone-protective properties. For example, one study revealed that hypoxia preconditioning significantly influenced the expression of immunomodulatory markers and exosome biogenesis in mesenchymal stromal cells (MSCs). ​ HLA-G mRNA expression showed a 10 ± 2-fold change at 24 h in WJ-MSCs and a 7 ± 2-fold change at 6 h in BM-MSCs, while AD-MSCs showed no significant response. IDO mRNA expression increased by 5 ± 3-fold at 6 h in WJ-MSCs and BM-MSCs, with AD-MSCs remaining unresponsive. ​ PGE-2 mRNA expression peaked at 55 ± 5-fold at 12 h in AD-MSCs and 20 ± 2-fold at 24 h in BM-MSCs, while WJ-MSCs showed no significant change. ​ Exosome biogenesis markers also showed differential responses: ALIX mRNA expression peaked at 11.29 ± 1.9-fold at 6 h in BM-MSCs and 14.05 ± 2.6-fold at 12 h in AD-MSCs, while Rab27b mRNA expression peaked at 19.1 ± 2.1-fold at 6 h in BM-MSCs and 69.78 ± 2.3-fold at 6 h in AD-MSCs. These findings highlight the tissue-specific and time-dependent effects of hypoxia on MSCs [228].

Liang et al. demonstrated that DMOG-preconditioned BMSC-derived exosomes (DMOG-MSC-Exos) accelerated endothelial cell migration and tube formation *in vitro* compared to standard MSC-Exos. In a rat critical-sized calvarial defect model, DMOG-MSC-Exos outperformed MSC-Exos and controls in bone regeneration, with larger vessel areas and increased CD31-positive staining, mediated through activation of the AKT/mTOR pathway [229]. Similarly, Wei et al. showed that hypoxia-preconditioned human umbilical cord MSC-derived exosomes (Hypo-Exos) significantly enhanced endothelial cell proliferation, migration, and tube formation *in vitro* versus normoxic MSC-Exos. In a mouse femoral fracture model, Hypo-Exos increased callus volume/tissue volume (CV/TV), vessel volume, and vessel number compared to Exos-treated groups, with miR-126 enriching the exosomes and promoting angiogenesis via SPRED1 targeting and Ras/Erk signaling activation [230].

Functionally engineered extracellular vesicles (FEEs) offer a complementary osteoinductive strategy. Chun et al. reported that BMP2-overexpressing HMSC-derived FEEs significantly increased osteogenic markers (BMP2, Runx2, Osterix/OSX, BMP9) and induced SMAD1/5/8 phosphorylation and luciferase activity in SMAD-specific reporter assays *in vitro*. In a rat calvarial defect model, μCT analysis showed a marked increase in bone volume (BV/TV) in BMP2 FEE-treated groups compared to control EVs and untreated defects. By 12 weeks, BMP2 FEEs nearly completely healed defects without ectopic bone formation, unlike rhBMP2, with histology confirming woven bone formation [231]. Collectively, these studies highlight the therapeutic potential of preconditioned or engineered MSC-derived exosomes and FEEs in promoting angiogenesis and osteogenesis for effective bone repair. These tailored, preconditioned exosomes represent a promising strategy for developing effective, systemic exosome-based therapies for OP, offering a targeted approach to bone regeneration and disease management.

### Improved Isolation: Yield, purity and cost-efficiency

Efficient and high-purity isolation of exosomes is essential for both research and therapeutic applications. Traditional ultracentrifugation (UC), while widely adopted, is often limited by lengthy processing times, low scalability, and contamination with non-vesicular proteins. To overcome these limitations, several advanced techniques have been developed to optimize exosome yield, purity, and cost-efficiency [232].

**Fig. 4:**
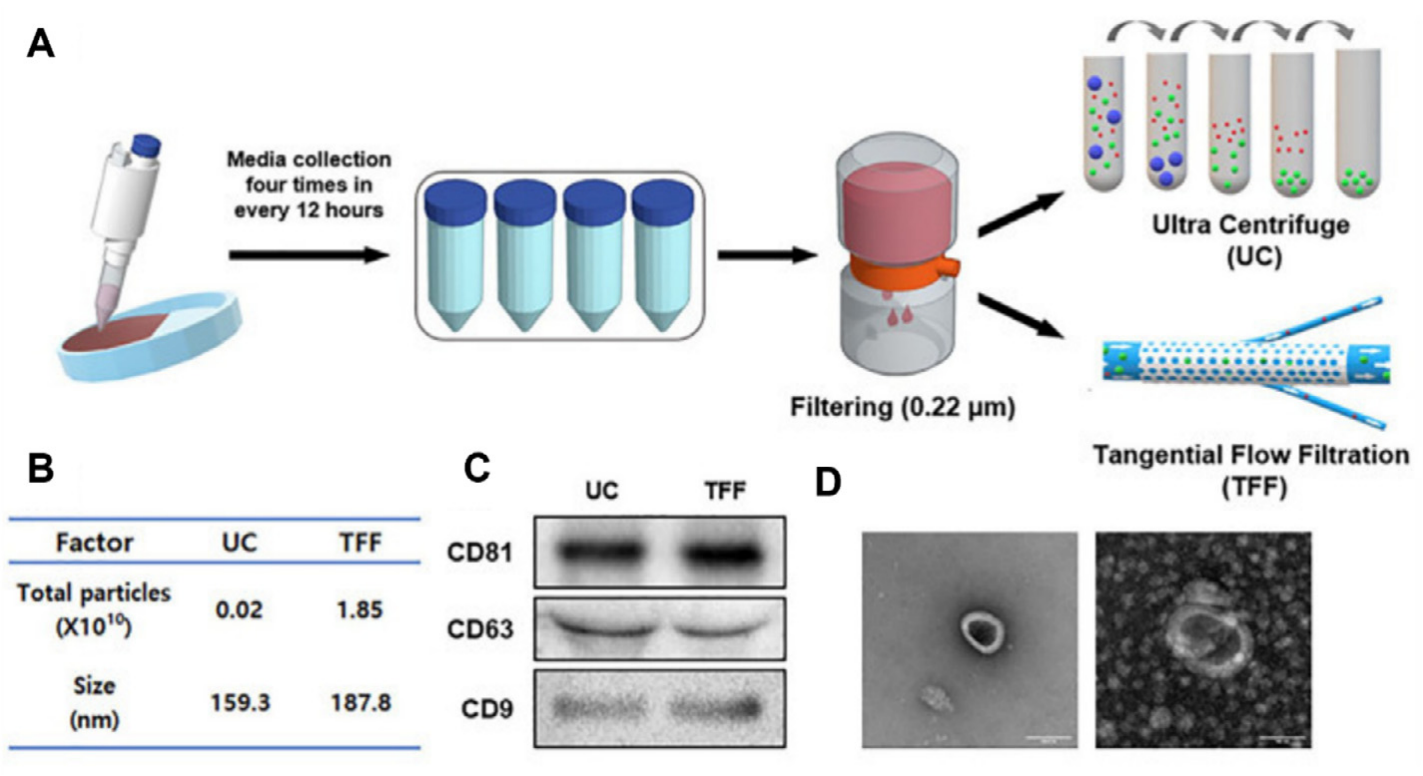
Comparison of exosome isolation methods and characterization of MSC-derived exosomes. (A) Schematic representation of the exosome isolation workflow from UCMSC-conditioned medium. Media collected four times at 12-hour intervals, followed by pre-filtration through a 0.22 μm membrane to remove large impurities. Exosomes were then isolated using two different methods: ultracentrifugation (UC) and tangential flow filtration (TFF).

One major direction has been the comparison between ultracentrifugation (UC) and tangential flow filtration (TFF) for isolating mesenchymal stem cell (MSC)-derived exosomes. In this approach, TFF, particularly when using a 500 kDa filter was shown to yield higher-purity exosomes more efficiently and rapidly than UC, while also being scalable and cost-effective. TFF yielded a significantly higher number of particles (1.85 × 10^1^⁰) compared to UC (0.02 × 10^1^⁰), with particle sizes of approximately 159.3 nm for UC and 187.8 nm for TFF (Fig. 4). Importantly, the study addressed a common concern in exosome production: contamination with fetal bovine serum (FBS)-derived exosomes. Here, ultrafiltration (UF) was identified as a superior depletion method for eliminating FBS contaminants without compromising MSC viability. The resulting purified exosomes demonstrated significantly enhanced angiogenic and wound healing effects, with their biological activity strongly influenced by exosome purity. Further validation using advanced characterization techniques, including FTIR spectroscopy and zeta potential analysis, confirmed structural and functional differences in exosome subpopulations. Collectively, this study proposed a standardized workflow combining TFF and UF-dFBS depletion as a robust and scalable strategy for generating high-purity MSC-derived exosomes suitable for clinical applications [233].

(B) Quantitative comparison of exosome yield and size between UC and TFF, determined by nanoparticle tracking analysis (NTA). (C) Western blot analysis confirming the presence of exosome-specific tetraspanins CD81, CD63, and CD9 in samples isolated by both methods, in accordance with MISEV2018 guidelines. (D) Transmission electron microscopy (TEM) images of exosomes showing the characteristic double-layered spherical morphology. Left: UC-derived exosomes; Right: TFF-derived exosomes. Scale bars: 100 nm. Licensed under CC BY-NC 4.0 (https://creativecommons.org/licenses/by-nc/4.0/).

In parallel, optimization efforts have also focused on exosomes isolated from human serum. A modified differential ultracentrifugation protocol was developed to reduce the traditional three-cycle process to two steps, while incorporating a 30 % sucrose cushion. This modification markedly improved the yield of exosomes while maintaining high purity by minimizing contamination from lipoproteins and RNA carrier proteins such as Ago2. Compared with the standard protocol, the modified approach demonstrated higher efficiency, better practicality, and maintained exosome integrity, thereby offering a more streamlined method for both scientific and clinical applications [234].

#### Role of RNA and immunomodulation in exosome-based therapies

The incorporation of RNA-based cargo and the modulation of immune responses represent emerging strategies that can significantly enhance the therapeutic potential of exosome-based treatments in OP.

#### RNa-loaded exosomes

RNA cargo-loaded exosomes offer an innovative way to modulate cellular behavior at the molecular level. Various RNA types, including miRNAs, siRNAs, and mRNAs, can be encapsulated within exosomes to regulate bone metabolism through targeted gene expression.

• **miRNAs**: These small non-coding RNAs regulate gene expression by binding to target mRNAs and inhibiting their translation. For OP, miRNAs that promote OB differentiation (e.g., miR-21 and miR-26a) or inhibit osteoclastogenesis (e.g., miR-155 and miR-146a) can be loaded into exosomes to enhance osteogenesis or prevent bone resorption, respectively [[235], [236], [237]].
• **siRNAs or shRNAs**: Exosomes can be bioengineered to deliver siRNAs targeting key osteoclastogenic genes, offering a precise strategy for treating OP. For example, exosomes loaded with RANK-specific siRNA inhibit OC formation and reduce bone resorption in osteoporotic models. Similarly, siRNAs directed against components of the NF-κB signaling pathway, such as p65 or IKKβ, inhibit OC differentiation by blocking key molecular signals, effectively mitigating bone loss [[238], [239], [240], [241]].
• **mRNA**-loaded exosomes carrying cargos, such as BMP-2 or Runx2 mRNA, directly upregulate osteogenic protein expression and activate key signaling pathways in OBs or MSCs, thereby stimulating osteogenic differentiation and accelerating bone regeneration in osteoporotic bone [242,243].

#### Immunomodulation

Immunomodulation via exosome-based approaches, particularly those involving Treg-derived exosomes, represents a novel strategy for targeting the chronic inflammation that contributes to OP. Tregs maintain immune homeostasis by suppressing pro-inflammatory cytokine production and facilitating tissue repair. Their exosomes carry immunosuppressive signals, including anti-inflammatory cytokines and regulatory miRNAs, which regulate immune responses. Treg-derived exosomes deliver immunoregulatory signals to osteoporotic bone tissue, modulating the local immune environment, reducing inflammation, and promoting bone healing. Additionally, these exosomes may interact with other immune cells, such as macrophages, to enhance the anti-inflammatory response, inhibit osteoclastogenesis, and stimulate osteogenesis [[244], [245], [246]]. This dual mechanism significantly enhances the therapeutic efficacies of exosome-based interventions in OP by promoting bone regeneration and regulating the pathological inflammation underlying bone loss.

Innovations in engineered exosomes, combination therapies incorporating biomaterials, immunomodulatory components, and RNA-based cargo are influencing advances in exosome-based therapies for OP. These strategies offer the potential for addressing current limitations and enhancing the therapeutic efficacy of exosomes, thereby facilitating their successful translation into clinical practice.

## Conclusion

### Bridging the gap between bone regeneration and osteoporosis

Exosomes represent an innovative therapeutic strategy for OP, offering novel strategies to address longstanding challenges in treating this prevalent and debilitating disease. As key mediators of cell-to-cell communication, exosomes deliver bioactive molecules and other signaling factors that modulate cellular processes essential for bone metabolism, including osteogenesis and osteoclastogenesis. These biological roles position exosomes as potent mediators of bone regeneration, with their ability to regulate OB differentiation, inhibit OC activity, and promote tissue repair, making them highly relevant for OP therapy.

OP, characterized by low bone mass, compromised bone microarchitecture, and increased fracture risk, is primarily attributed to an imbalance between osteoblastic bone formation and osteoclastic bone resorption. Traditional treatments, such as BPs, denosumab, and hormone replacement therapy, target the inhibition of bone resorption or the stimulation of bone formation; however, none offer a comprehensive solution that fully restores bone health. In contrast, exosomes represent a novel strategy capable of overcoming many limitations of current therapies. These natural delivery systems efficiently transport specific therapeutic molecules that influence the functions of both OBs and OCs.

Furthermore, exosomes derived from various sources such as MSCs, OBs, OCs, and immune cells offer a multifaceted approach to addressing OP. MSC-derived exosomes demonstrate the potential to promote osteogenesis and stimulate bone formation, while OC-derived exosomes may regulate bone resorption. Loading exosomes with osteogenic factors, miRNAs, or RNA-based therapeutics (e.g., siRNAs or mRNAs) can enhance their therapeutic efficacy, enabling targeted and personalized approaches for OP.

Additionally, engineered exosomes offer significant benefits due to their modifiability, facilitating targeting specificity, enhanced cargo delivery, and increased stability. Combining exosomes with other therapeutic strategies, such as immunomodulatory therapies (e.g., Treg-based therapies), further enhances their potential as an advanced treatment modality for OP. Integrating the regenerative capabilities of exosomes with complementary approaches enables more effective management of the complex, multifactorial nature of OP, offering a promising path toward durable bone regeneration.

Despite their immense potential, significant challenges remain before exosome-based therapies can be successfully translated into clinical practice. Issues related to the isolation, characterization, and efficient delivery of exosomes, along with their targeting specificity to osteoporotic bone tissue, require resolution. The lack of large-scale clinical studies and standardized protocols for exosome production and application constitutes a significant barrier. However, as the understanding of exosome biology and its role in bone regeneration advances, solutions to these challenges are expected to emerge.

### Call for further research

To fully realize the therapeutic potential of exosomes in OP, continued research remains essential. While significant progress has been made in understanding exosome biology and its applications in bone regeneration, critical knowledge gaps persist. Standardizing methods for exosome isolation and characterization should be prioritized. Given the inherent heterogeneity of exosomes, optimizing techniques for consistent isolation, purification, and characterization across different sources and experimental models is essential to ensure reproducibility and reliability in therapeutic applications.

Additionally, the mechanisms underlying exosome uptake by target cells, particularly in osteoporotic bone tissue, remain unclear. Detailed studies investigating how exosomes navigate biological barriers, interact with specific cell types in the bone microenvironment, and release their cargo are necessary to optimize their *in vivo* efficacy. Furthermore, examining the stability and biodistribution of exosomes, particularly regarding systemic circulation and bone tissue localization, is crucial for improving their therapeutic outcomes.

Another critical area for further research involves enhancing exosome cargo loading and release strategies. While natural exosomes carry various bioactive molecules, selectively loading specific RNA species, proteins, or small molecules that modulate osteogenesis or osteoclastogenesis could facilitate more targeted and effective treatments. Future studies should focus on improving methods for efficient cargo incorporation, ensuring therapeutic agents retain functionality upon delivery, and developing controlled-release systems with long-term therapeutic effects.

Moreover, larger and more diverse preclinical and clinical studies are essential to establish the safety, efficacy, and optimal dosages of exosome-based therapies for OP. Given that exosomes derived from different cell types may exhibit varied effects, identifying the most effective exosome sources for OP treatment is crucial. Clinical trials evaluating the long-term effects of exosome-based therapies, their potential synergistic interactions with existing treatments, and their safety profiles are critical for advancing these therapies into routine clinical practice.

As the potential of engineered exosomes to overcome existing therapeutic challenges advances, interdisciplinary collaboration among researchers in stem cell biology, materials science, immunology, and clinical medicine becomes increasingly necessary. Combining expertise from these fields will enable more integrated and effective approaches for addressing the multifaceted challenges of OP.

In conclusion, exosomes and exosome-mimetic vesicles collectively address the core drivers of OP by (i) promoting osteogenesis, (ii) restraining osteoclastogenesis, and (iii) re-balancing the inflammatory/immune milieu that sustains bone loss. Evidence shows osteogenic and immunomodulatory miRNA cargo (e.g., miR-26a, miR-335, miR-503-5p, miR-146a, miR-21, miR-150-5p) elevates RUNX2/ALP and YAP1-dependent programs, reduces RANKL–NF-κB signaling, and improves trabecular microarchitecture *in vivo*, including with endothelial- and Treg-derived exosomes that add angiogenic and M2-polarizing benefits. Importantly, OP subtype biology implies therapeutic tailoring: anti-resorptive, immunomodulatory cargo is most relevant for postmenopausal OP, whereas senile OP benefits from rejuvenation-oriented, pro-osteogenic/angiogenic and anti-senescence payloads. Emerging bioengineering strategies bone-targeting ligands, hybrid vesicles, and stimuli-responsive depots now enable selective accumulation in osteoporotic bone and sustained, on-site release, moving the field from proof-of-concept to translational feasibility. Altogether, exosome-based therapeutics are poised as disease-modifying candidates that could complement and in targeted contexts surpass current anti-resorptives and anabolics by concurrently restoring bone formation–resorption coupling and immune homeostasis.

### Future directions: translating exosome-based therapies into clinical practice

The next frontier in exosome-based OP therapy lies in precision engineering, integration with advanced technologies, and development of clinically viable strategies. Future research should focus on the following innovative directions:

1. **Subtype-specific and personalized exosome therapy**

Future OP therapies must address distinct pathophysiological signatures of PMOP (estrogen deficiency, NF-κB/RANKL-driven osteoclastogenesis) and SOP (stem cell senescence, oxidative stress). Multi-omics profiling (miRNome, proteome) can guide personalized exosome designs:

• PMOP-focused exosomes: Enriched with miR-155, miR-503-5p, and miR-146a to inhibit NF-κB and RANKL signaling, reducing OC activity.
• SOP-focused exosomes: Loaded with miR-29a, miR-21-5p, and miR-335, which upregulate RUNX2, BMP2, and COL1A1 to restore osteoblastogenesis and angiogenesis.
• AI-based cargo optimization: Predictive algorithms can optimize miRNA cocktails (e.g., miR-26a + miR-21 + miR-378a) for specific patient profiles.

2. **Theranostic and smart-release exosome systems**

Next-generation platforms should combine therapy with real-time tracking and stimuli-responsive release:

• Theranostic exosomes conjugated with Mn^2+^ NPs for MRI visualization and osteogenic cargo (e.g., SIRT1 agonist SRT2104, RUNX2 mRNA).
• ROS-responsive hydrogels encapsulating exosomes to release miRNA payloads (miR-21, miR-29b) in high oxidative-stress osteoporotic niches.
• pH-sensitive coatings for targeted release in resorption lacunae, delivering inhibitors such as siRNA-RANK or siRNA-MMP9 to suppress OC activity.

3. **Combination therapies and hybrid constructs**

Integrating exosomes with small molecules, biologics, and biomaterials can enhance multi-modal effects:

• Senolytic-exosome hybrids: Combine exosomes enriched with anti-senescence factors (miR-34a inhibitors) and Quercetin/Dasatinib for rejuvenating aged osteocytes in SOP.
• Biomaterial synergy: Embed exosomes loaded with BMP2 mRNA, VEGF-A, or miR-126 into 3D-printed HA scaffolds for simultaneous osteogenesis and angiogenesis.

4. **Advanced bioengineering for bone targeting**

Bone-targeted exosomes can dramatically improve localization and therapeutic index:

• Surface modification with (Asp-Ser-Ser)_6_ peptide (DSS_6_) or alendronate conjugation for hydroxyapatite binding and selective bone accumulation.
• Exosome–antibody conjugates: Surface functionalization with anti-RANK or anti-integrin αvβ3 antibodies for targeted OC inhibition.
• Dual-ligand functionalization: DSS_6_ for mineralized bone + RGD peptide for integrin-mediated osteoblast uptake.
• Click-chemistry approaches to stably link osteoanabolic ligands (e.g., periostin-binding peptides) onto exosome membranes.

5. **GMP-compliant biomanufacturing and scale-up**

To transition into clinical use:

• Employ continuous perfusion bioreactors for scalable exosome production.
• Use microfluidic electroporation for high-efficiency loading of therapeutic RNAs (e.g., miR-503-5p, miR-21, siRANKL).
• Develop synthetic exosome mimetics with controlled lipid composition and customizable molecular cargo for regulatory compliance.
6. **Next-generation preclinical models and clinical design**
• Osteoporotic organ-on-chip platforms replicating bone-vascular-immune interactions for high-throughput screening of engineered exosomes.
• Adaptive clinical trial designs incorporating exosome-based biomarkers (e.g., circulating miR-503 or miR-21 levels) for stratifying responders and optimizing dose.

**Fig. 5:**
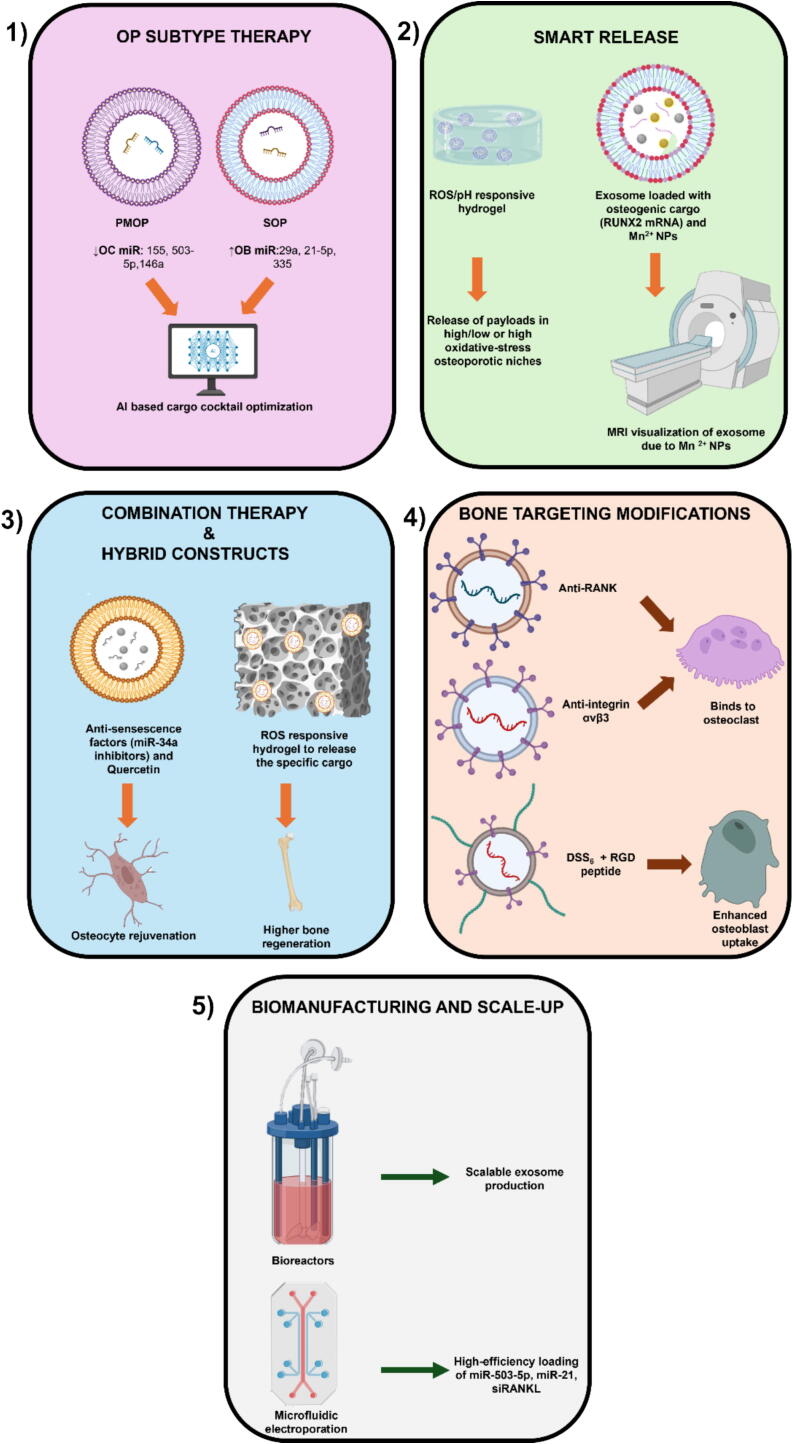
Future perspectives in exosome-based therapeutics for osteoporosis.

Taken together, these strategies emphasize that the successful translation of exosome-based therapeutics for OP will depend on integrating biological insight with engineering innovation, rigorous standardization, and clinical validation (Fig. 5). With coordinated interdisciplinary efforts, exosome-based interventions have the potential not only to complement but also to redefine current OP management by offering durable, targeted, and disease-modifying therapies.

Schematic representation of emerging strategies in exosome engineering and application for OP therapy. (1) **OP subtype therapy:** Personalized exosome formulations tailored to PMOP or SOP using AI-guided optimization of miRNA cargo. (2) **Smart release:** Stimuli-responsive systems such as ROS/pH-sensitive hydrogels and exosomes loaded with osteogenic payloads (e.g., RUNX2 mRNA, Mn^2+^ nanoparticles) enabling controlled release and theranostic imaging. (3) **Combination therapy and hybrid constructs:** Integration of exosomes with senolytic agents or biomaterials to rejuvenate osteocytes and enhance bone regeneration. (4) **Bone-targeting modifications:** Functionalization with antibodies (anti-RANK, anti-integrin αvβ3), peptides (RGD, DSS_6_), or ligands to selectively inhibit osteoclasts and improve osteoblast uptake. (5) **Biomanufacturing and scale-up:** GMP-compatible production using bioreactors and microfluidic electroporation for high-efficiency RNA loading and clinical translation. Created with BioRender.

## Consent for publication

Not applicable.

## Author’s contributions

The review topic was conceived and designed by P.D.A., Y.A., and S.L. The manuscript was drafted by P.D.A., and A.B., and it was revised and critically edited by Y.A., and S.L.

## Ethics approval and consent to participate

Not applicable.

## Declaration of competing interest

The authors declare that they have no known competing financial interests or personal relationships that could have appeared to influence the work reported in this paper.
